# Tests for differential gene expression using weights in oligonucleotide microarray experiments

**DOI:** 10.1186/1471-2164-7-33

**Published:** 2006-02-22

**Authors:** Pingzhao Hu, Joseph Beyene, Celia MT Greenwood

**Affiliations:** 1 Program in Genetics and Genomic Biology, The Hospital for Sick Children Research Institute, 15-706 TMDT, 101 College Street, Toronto, ON, M5G 1L7, Canada; 2 Department of Public Health Sciences, University of Toronto, Health Sciences Building, 155 College St, Toronto, ON, M5T 3M7, Canada; 3 Program in Population Health Sciences, The Hospital for Sick Children Research Institute, 555 University Ave, Toronto, ON, M5G 1X8, Canada

## Abstract

**Background:**

Microarray data analysts commonly filter out genes based on a number of ad hoc criteria prior to any high-level statistical analysis. Such ad hoc approaches could lead to conflicting conclusions with no clear guidance as to which method is most likely to be reproducible. Furthermore, the number of tests performed with concomitant inflation in type I error also plagues the statistical analysis of microarray data, since the number of tested quantities in a study significantly affects the family-wise error rate. It would, therefore, be very useful to develop and adopt strategies that allow quantification of the quality of each probeset, to filter out or give little credence to low-quality or unexpressed probesets, and to incorporate these strategies into gene selection within a multiple testing framework.

**Results:**

We have proposed a unified scheme for filtering and gene selection. For Affymetrix gene expression microarrays, we developed new methods for measuring the reliability of a particular probeset in a single array, and we used these to develop measures for a set of arrays. These measures are then used as weights in standard t-statistic calculations, and are incorporated into the multiple testing procedures. We demonstrated the advantages of our methods using simulated data, publicly available spiked-in data as well as data comparing normal muscle to muscle from patients with Duchenne muscular dystrophy (DMD), in which a set of truly differentially expressed genes is known.

**Conclusion:**

Our quality measures provide convenient ways to search for individual genes of high quality. The quality weighting strategies we proposed for testing differential gene expression have demonstrable improvement on the traditional filtering methods, the standard t-statistic and a regularized t-statistic in Affymetrix data analysis.

## Background

Affymetrix GeneChip™ microarrays are used to measure gene expression for thousands of transcripts simultaneously. Each transcript is measured by 11–20 probesets, where a probeset consists of two almost identical sequences of length 25 bp. One member of the pair is the perfect match (PM) probe, where the sequence is the exact complement of a section of the mRNA. The other member of the pair, the mismatch probe (MM), is identical to the PM probe except for the nucleotide in 13^th ^position and is intended to measure and control for non-specific binding signals. The accuracy and sensitivity of the measurement for any one gene or EST depends on the uniqueness and the binding properties of the probes.

It is well known that most probesets perform consistently and reliably, in that similar estimates of expression are obtained from two replicates of an experiment. However, in many cases, the signals from a probeset can be hard to interpret. There may be substantial variability across the probesets in the estimated level of expression, or in the PM-MM differences. PM signals can be smaller than MM signals suggesting high levels of non-specific binding. It is also well known that a large proportion of the genes are expressed in only a few tissues or at a particular developmental stage, and hence many of the genes are not expected to have a measurable transcribed product. Su et al. [1] have estimated that in most cases, only 30–40% of the genome will be expressed in any one tissue. In such situations, the probesets give measurements for PM and MM that fluctuate near the lower detectable limit.

The latest release for the Affymetrix GeneChip Expression Analysis Platform, GeneChip Human Genome U133 Plus 2.0 Array [2], provides comprehensive coverage of the transcribed human genome on a single array. The array includes more than 54,000 probesets with 38,500 well-characterized human genes. When analyzing such a large number of genes, the adjustment of significance levels through multiple testing procedures such as the Bonferroni method [3], or Benjamini and Hochberg [4], may be dramatic enough to make it very difficult to identify differentially expressed genes. However, if we knew which transcripts were either not expressed in the tissue under study, or were measured unreliably due to poor probe specificity, we could exclude these transcripts from the analysis and pay a smaller penalty for multiple testing.

In 2001, Affymetrix produced a new algorithm for summarizing the results of a probeset, and the algorithm includes a detection p-value, which represents the probability that the probeset (gene) expression is above zero (i.e., turned on), and measured reliably and consistently across the probe. In addition, "Present/Marginal/Absent" (P/M/A) calls for each transcript are based on the detection p-value together with thresholds that can be altered by the user [5]. The calls are often used as filters to keep genes whose transcripts are detectable in a particular experiment [6-8], and this filtering concept has been integrated into some software such as dChip [9,10]. For example, Blalock, et al. [7] removed a probeset from further statistical analysis if there were > = 60% absent calls, for that probeset, in at least one treatment group. However, the stringency of the filtering procedure can strongly affect downstream analyses and the final results. Too much filtering may exclude some important genes, whereas too little filtering will reduce power by increasing the number of tests performed. Moreover, it is possible that the statistical error introduced by imperfect filtering criteria makes the overall error worse. Pounds and Cheng [11] recently argued that it is better to define gene filters based on the detection p-value than on the calls, so that more of the available information about probeset reliability can be used. They developed two pooled filters for each probeset based on detection p-values.

An alternative strategy to filtering is to treat probe-specific variability as a quality index to measure the reliability of each probeset. Although many summaries of Affymetrix GeneChip expression have been proposed (e.g. MAS5 [5], dChip [9,10], RMA [12], PLIER [13] and gcRMA [14]), there have been few studies on quality measures for Affymetrix GeneChip expression data. In previous studies, typically, once a filtering decision was made, the estimated intensities of each probeset were considered to be equally well measured, so that probesets with highly variable signals were considered as reliable as probesets with inconsistent signals. However, probesets that detect transcripts expressed at a very high level would be expected to show a more robust signal with greater quality than probesets that are performing poorly or detecting very low level transcripts.

Seo et al. [15] used the detection p-values to develop weighted Pearson correlations between expression measurements from two different arrays. The weight was defined to be a function of the two detection p-values for each probeset. They incorporated these weighted correlations into unsupervised clustering analysis, with the goal of choosing the best algorithm for summarizing across the probesets.

In this study, we focus on the problem of testing for differential expression between groups of arrays, while adjusting for multiple testing in a weighted framework. We redefine some of the widely used filtering methods [6-9] and also propose some new methods for measuring the quality of a particular probeset in a single array, in an experimental group of arrays, and in an entire study. These measures are then used as weights in t-statistic calculations, and are incorporated into the multiple testing procedures. We applied these methods to a dataset of expression in muscle tissue [16], Choe's spiked-in data [17] and simulated data. One of our quality measures for an experimental group is based on the measure developed very recently by Pounds and Cheng [11]; however, we go further in combining measures across groups, and in discussing the usefulness of these measures when testing for differential gene expression.

## Results

### Test statistics and quality measures

We propose a weighting paradigm for including quality measures into analysis when testing for differential expression. Suppose that an unweighted test statistic for gene *g *is represented by *t*_*g*_, and that the quality measure is called *Q*_*g*_. We propose to evaluate the significance of gene *g *using the weighted test statistic tg*
 MathType@MTEF@5@5@+=feaafiart1ev1aaatCvAUfKttLearuWrP9MDH5MBPbIqV92AaeXatLxBI9gBaebbnrfifHhDYfgasaacH8akY=wiFfYdH8Gipec8Eeeu0xXdbba9frFj0=OqFfea0dXdd9vqai=hGuQ8kuc9pgc9s8qqaq=dirpe0xb9q8qiLsFr0=vr0=vr0dc8meaabaqaciaacaGaaeqabaqabeGadaaakeaacqWG0baDdaqhaaWcbaGaem4zaCgabaGaeiOkaOcaaaaa@307D@ = *t*_*g*_*Q*_*g*_. The impact of the weighting on the pattern of results, across all genes, is then taken into account when calculating significance adjusted for multiple testing, or when estimating the false discovery rate. Conceptually, giving a low weight, *Q*_*g*_≈0, to a particular gene can be thought of as excluding that gene from consideration. Therefore, our modifications to multiple-testing methods are based on adjusting the number of genes tested, based on the quality measures.

Measure *Q*_*g *_must represent a summary across treatment groups *w *∈ {1 ...*W*}. We propose

Qg=max⁡w∈{1…W}[Rgw],     (1)
 MathType@MTEF@5@5@+=feaafiart1ev1aaatCvAUfKttLearuWrP9MDH5MBPbIqV92AaeXatLxBI9gBaebbnrfifHhDYfgasaacH8akY=wiFfYdH8Gipec8Eeeu0xXdbba9frFj0=OqFfea0dXdd9vqai=hGuQ8kuc9pgc9s8qqaq=dirpe0xb9q8qiLsFr0=vr0=vr0dc8meaabaqaciaacaGaaeqabaqabeGadaaakeaacqWGrbqudaWgaaWcbaGaem4zaCgabeaakiabg2da9iGbc2gaTjabcggaHjabcIha4naaBaaaleaacqWG3bWDcqGHiiIZcqGG7bWEcqaIXaqmcqWIMaYscqWGxbWvcqGG9bqFaeqaaOGaei4waSLaemOuai1aa0baaSqaaiabdEgaNbqaaiabdEha3baakiabc2faDjabcYcaSiaaxMaacaWLjaWaaeWaaeaacqaIXaqmaiaawIcacaGLPaaaaaa@4959@

where Rgw
 MathType@MTEF@5@5@+=feaafiart1ev1aaatCvAUfKttLearuWrP9MDH5MBPbIqV92AaeXatLxBI9gBaebbnrfifHhDYfgasaacH8akY=wiFfYdH8Gipec8Eeeu0xXdbba9frFj0=OqFfea0dXdd9vqai=hGuQ8kuc9pgc9s8qqaq=dirpe0xb9q8qiLsFr0=vr0=vr0dc8meaabaqaciaacaGaaeqabaqabeGadaaakeaacqWGsbGudaqhaaWcbaGaem4zaCgabaGaem4DaChaaaaa@30D4@ is a treatment-group specific measure of quality. By using the maximum value across groups in equation (1), a gene that is clearly expressed in one or more groups will be considered as a gene measured with high quality.

We examine the performance of several choices for the group-specific quality measure Rgw
 MathType@MTEF@5@5@+=feaafiart1ev1aaatCvAUfKttLearuWrP9MDH5MBPbIqV92AaeXatLxBI9gBaebbnrfifHhDYfgasaacH8akY=wiFfYdH8Gipec8Eeeu0xXdbba9frFj0=OqFfea0dXdd9vqai=hGuQ8kuc9pgc9s8qqaq=dirpe0xb9q8qiLsFr0=vr0=vr0dc8meaabaqaciaacaGaaeqabaqabeGadaaakeaacqWGsbGudaqhaaWcbaGaem4zaCgabaGaem4DaChaaaaa@30D4@. In particular, Rgw
 MathType@MTEF@5@5@+=feaafiart1ev1aaatCvAUfKttLearuWrP9MDH5MBPbIqV92AaeXatLxBI9gBaebbnrfifHhDYfgasaacH8akY=wiFfYdH8Gipec8Eeeu0xXdbba9frFj0=OqFfea0dXdd9vqai=hGuQ8kuc9pgc9s8qqaq=dirpe0xb9q8qiLsFr0=vr0=vr0dc8meaabaqaciaacaGaaeqabaqabeGadaaakeaacqWGsbGudaqhaaWcbaGaem4zaCgabaGaem4DaChaaaaa@30D4@ = 1.0 leads, after using equation (1), to a measure Qg0
 MathType@MTEF@5@5@+=feaafiart1ev1aaatCvAUfKttLearuWrP9MDH5MBPbIqV92AaeXatLxBI9gBaebbnrfifHhDYfgasaacH8akY=wiFfYdH8Gipec8Eeeu0xXdbba9frFj0=OqFfea0dXdd9vqai=hGuQ8kuc9pgc9s8qqaq=dirpe0xb9q8qiLsFr0=vr0=vr0dc8meaabaqaciaacaGaaeqabaqabeGadaaakeaacqWGrbqudaqhaaWcbaGaem4zaCgabaGaeGimaadaaaaa@3049@ that is, in fact, no quality filtering: all genes are included in the analysis at full weight. The common practice of analyzing only genes with present calls can be based on {Rgw
 MathType@MTEF@5@5@+=feaafiart1ev1aaatCvAUfKttLearuWrP9MDH5MBPbIqV92AaeXatLxBI9gBaebbnrfifHhDYfgasaacH8akY=wiFfYdH8Gipec8Eeeu0xXdbba9frFj0=OqFfea0dXdd9vqai=hGuQ8kuc9pgc9s8qqaq=dirpe0xb9q8qiLsFr0=vr0=vr0dc8meaabaqaciaacaGaaeqabaqabeGadaaakeaacqWGsbGudaqhaaWcbaGaem4zaCgabaGaem4DaChaaaaa@30D4@ = 1.0 when all arrays in group *w *have present calls, and Rgw
 MathType@MTEF@5@5@+=feaafiart1ev1aaatCvAUfKttLearuWrP9MDH5MBPbIqV92AaeXatLxBI9gBaebbnrfifHhDYfgasaacH8akY=wiFfYdH8Gipec8Eeeu0xXdbba9frFj0=OqFfea0dXdd9vqai=hGuQ8kuc9pgc9s8qqaq=dirpe0xb9q8qiLsFr0=vr0=vr0dc8meaabaqaciaacaGaaeqabaqabeGadaaakeaacqWGsbGudaqhaaWcbaGaem4zaCgabaGaem4DaChaaaaa@30D4@ = 0 otherwise}. We will denote this as Qgcall
 MathType@MTEF@5@5@+=feaafiart1ev1aaatCvAUfKttLearuWrP9MDH5MBPbIqV92AaeXatLxBI9gBaebbnrfifHhDYfgasaacH8akY=wiFfYdH8Gipec8Eeeu0xXdbba9frFj0=OqFfea0dXdd9vqai=hGuQ8kuc9pgc9s8qqaq=dirpe0xb9q8qiLsFr0=vr0=vr0dc8meaabaqaciaacaGaaeqabaqabeGadaaakeaacqWGrbqudaqhaaWcbaGaem4zaCgabaGaem4yamMaemyyaeMaemiBaWMaemiBaWgaaaaa@34B7@.

Instead of using the Affymetrix present/absent calls, more sensitivity can be gained by basing Rgw
 MathType@MTEF@5@5@+=feaafiart1ev1aaatCvAUfKttLearuWrP9MDH5MBPbIqV92AaeXatLxBI9gBaebbnrfifHhDYfgasaacH8akY=wiFfYdH8Gipec8Eeeu0xXdbba9frFj0=OqFfea0dXdd9vqai=hGuQ8kuc9pgc9s8qqaq=dirpe0xb9q8qiLsFr0=vr0=vr0dc8meaabaqaciaacaGaaeqabaqabeGadaaakeaacqWGsbGudaqhaaWcbaGaem4zaCgabaGaem4DaChaaaaa@30D4@ on the actual detection p-values, *q*_*giw *_for gene *g*, array *i*, and group *w*. Since a highly-expressed gene corresponds to a small detection p-value, we propose Rgw
 MathType@MTEF@5@5@+=feaafiart1ev1aaatCvAUfKttLearuWrP9MDH5MBPbIqV92AaeXatLxBI9gBaebbnrfifHhDYfgasaacH8akY=wiFfYdH8Gipec8Eeeu0xXdbba9frFj0=OqFfea0dXdd9vqai=hGuQ8kuc9pgc9s8qqaq=dirpe0xb9q8qiLsFr0=vr0=vr0dc8meaabaqaciaacaGaaeqabaqabeGadaaakeaacqWGsbGudaqhaaWcbaGaem4zaCgabaGaem4DaChaaaaa@30D4@ = 1 - q¯gw
 MathType@MTEF@5@5@+=feaafiart1ev1aaatCvAUfKttLearuWrP9MDH5MBPbIqV92AaeXatLxBI9gBaebbnrfifHhDYfgasaacH8akY=wiFfYdH8Gipec8Eeeu0xXdbba9frFj0=OqFfea0dXdd9vqai=hGuQ8kuc9pgc9s8qqaq=dirpe0xb9q8qiLsFr0=vr0=vr0dc8meaabaqaciaacaGaaeqabaqabeGadaaakeaacuWGXbqCgaqeamaaBaaaleaacqWGNbWzcqWG3bWDaeqaaaaa@3129@, where q¯gw
 MathType@MTEF@5@5@+=feaafiart1ev1aaatCvAUfKttLearuWrP9MDH5MBPbIqV92AaeXatLxBI9gBaebbnrfifHhDYfgasaacH8akY=wiFfYdH8Gipec8Eeeu0xXdbba9frFj0=OqFfea0dXdd9vqai=hGuQ8kuc9pgc9s8qqaq=dirpe0xb9q8qiLsFr0=vr0=vr0dc8meaabaqaciaacaGaaeqabaqabeGadaaakeaacuWGXbqCgaqeamaaBaaaleaacqWGNbWzcqWG3bWDaeqaaaaa@3129@ is the mean detection p-value for group *w*, leading to a quality measure that we call Qgmeanp
 MathType@MTEF@5@5@+=feaafiart1ev1aaatCvAUfKttLearuWrP9MDH5MBPbIqV92AaeXatLxBI9gBaebbnrfifHhDYfgasaacH8akY=wiFfYdH8Gipec8Eeeu0xXdbba9frFj0=OqFfea0dXdd9vqai=hGuQ8kuc9pgc9s8qqaq=dirpe0xb9q8qiLsFr0=vr0=vr0dc8meaabaqaciaacaGaaeqabaqabeGadaaakeaacqWGrbqudaqhaaWcbaGaem4zaCgabaGaemyBa0MaemyzauMaemyyaeMaemOBa4MaemiCaahaaaaa@362A@. The mean may not give enough weight to small p-values, therefore we also investigated two measures that are based on parametric model assumptions for the distribution of detection p-values. Firstly, we assumed that -log(*q*_*giw*_) follow an exponential distribution with group-specific mean *λ*_*qw*_. Under this distribution, the probability that the detection p-values will be smaller than a threshold *ν *can be written as Rgw=exp⁡(λ^gwlog⁡ν)
 MathType@MTEF@5@5@+=feaafiart1ev1aaatCvAUfKttLearuWrP9MDH5MBPbIqV92AaeXatLxBI9gBaebbnrfifHhDYfgasaacH8akY=wiFfYdH8Gipec8Eeeu0xXdbba9frFj0=OqFfea0dXdd9vqai=hGuQ8kuc9pgc9s8qqaq=dirpe0xb9q8qiLsFr0=vr0=vr0dc8meaabaqaciaacaGaaeqabaqabeGadaaakeaacqWGsbGudaqhaaWcbaGaem4zaCgabaGaem4DaChaaOGaeyypa0JagiyzauMaeiiEaGNaeiiCaaNaeiikaGccciGaf83UdWMbaKaadaWgaaWcbaGaem4zaCMaem4DaChabeaakiGbcYgaSjabc+gaVjabcEgaNjab=17aUjabcMcaPaaa@426A@, with corresponding Qgexp⁡
 MathType@MTEF@5@5@+=feaafiart1ev1aaatCvAUfKttLearuWrP9MDH5MBPbIqV92AaeXatLxBI9gBaebbnrfifHhDYfgasaacH8akY=wiFfYdH8Gipec8Eeeu0xXdbba9frFj0=OqFfea0dXdd9vqai=hGuQ8kuc9pgc9s8qqaq=dirpe0xb9q8qiLsFr0=vr0=vr0dc8meaabaqaciaacaGaaeqabaqabeGadaaakeaacqWGrbqudaqhaaWcbaGaem4zaCgabaGagiyzauMaeiiEaGNaeiiCaahaaaaa@338F@ following from equation (1).

Although, under the null hypothesis of no signal, the detection p-values can be expected to follow a uniform distribution (so that -log(*q*) is exponential with mean 1.0), when there is a signal, the p-value distribution is better described by the two-parameter Beta distribution. However, detection p-values are based on rank tests and there is often little variability in the p-values across arrays for highly expressed genes. This leads to difficulties in estimating two parameters. Pounds and Cheng [11] proposed a one-parameter Beta distribution for detection p-values, so that Rgw=p(qgiw≤ν)=∫0νBeta(q;a^gw)dq=νa^gw
MathType@MTEF@5@5@+=feaafiart1ev1aaatCvAUfKttLearuWrP9MDH5MBPbIqV92AaeXatLxBI9gBaebbnrfifHhDYfgasaacH8akY=wiFfYdH8Gipec8Eeeu0xXdbba9frFj0=OqFfea0dXdd9vqai=hGuQ8kuc9pgc9s8qqaq=dirpe0xb9q8qiLsFr0=vr0=vr0dc8meaabaqaciaacaGaaeqabaqabeGadaaakeaacqWGsbGudaqhaaWcbaGaem4zaCgabaGaem4DaChaaOGaeyypa0JaemiCaaNaeiikaGIaemyCae3aaSbaaSqaaiabdEgaNjabdMgaPjabdEha3bqabaGccqGHKjYOiiGacqWF9oGBcqGGPaqkcqGH9aqpdaWdXbqaaiabdkeacjabdwgaLjabdsha0jabdggaHjabcIcaOiabdghaXjabcUda7iqbdggaHzaajaWaaSbaaSqaaiabdEgaNjabdEha3bqabaGccqGGPaqkcqWGKbazcqWGXbqCaSqaaiabicdaWaqaaiab=17aUbqdcqGHRiI8aOGaeyypa0Jae8xVd42aaWbaaSqabeaacuWGHbqygaqcaiabdEgaNjabdEha3baaaaa@5BB8@, and we incorporate this measure into our corresponding across-group quality measure Qgbeta
 MathType@MTEF@5@5@+=feaafiart1ev1aaatCvAUfKttLearuWrP9MDH5MBPbIqV92AaeXatLxBI9gBaebbnrfifHhDYfgasaacH8akY=wiFfYdH8Gipec8Eeeu0xXdbba9frFj0=OqFfea0dXdd9vqai=hGuQ8kuc9pgc9s8qqaq=dirpe0xb9q8qiLsFr0=vr0=vr0dc8meaabaqaciaacaGaaeqabaqabeGadaaakeaacqWGrbqudaqhaaWcbaGaem4zaCgabaGaemOyaiMaemyzauMaemiDaqNaemyyaegaaaaa@34B7@.

For comparison, two very different test statistics were also used: (1) Weights based on the detection p-values were incorporated directly in the calculation of group-specific means and standard deviations – we refer to this test statistic as Qgwe
 MathType@MTEF@5@5@+=feaafiart1ev1aaatCvAUfKttLearuWrP9MDH5MBPbIqV92AaeXatLxBI9gBaebbnrfifHhDYfgasaacH8akY=wiFfYdH8Gipec8Eeeu0xXdbba9frFj0=OqFfea0dXdd9vqai=hGuQ8kuc9pgc9s8qqaq=dirpe0xb9q8qiLsFr0=vr0=vr0dc8meaabaqaciaacaGaaeqabaqabeGadaaakeaacqWGrbqudaqhaaWcbaGaem4zaCgabaGaem4DaCNaemyzaugaaaaa@3225@ to follow our naming convention, although no quality measure is defined; (2) In addition, we used the local pooled error (LPE) test [18], which is based on pooling errors within genes and between replicate arrays for genes in which expression values are similar. This test is referred to as QgLPE
 MathType@MTEF@5@5@+=feaafiart1ev1aaatCvAUfKttLearuWrP9MDH5MBPbIqV92AaeXatLxBI9gBaebbnrfifHhDYfgasaacH8akY=wiFfYdH8Gipec8Eeeu0xXdbba9frFj0=OqFfea0dXdd9vqai=hGuQ8kuc9pgc9s8qqaq=dirpe0xb9q8qiLsFr0=vr0=vr0dc8meaabaqaciaacaGaaeqabaqabeGadaaakeaacqWGrbqudaqhaaWcbaGaem4zaCgabaGaemitaWKaemiuaaLaemyraueaaaaa@32B8@. Unweighted multiple testing corrections were implemented for these two tests. Table 1 summarizes the different test statistics and quality measures used in the paper.

### Analysis of simulated data

**Figure 1:**
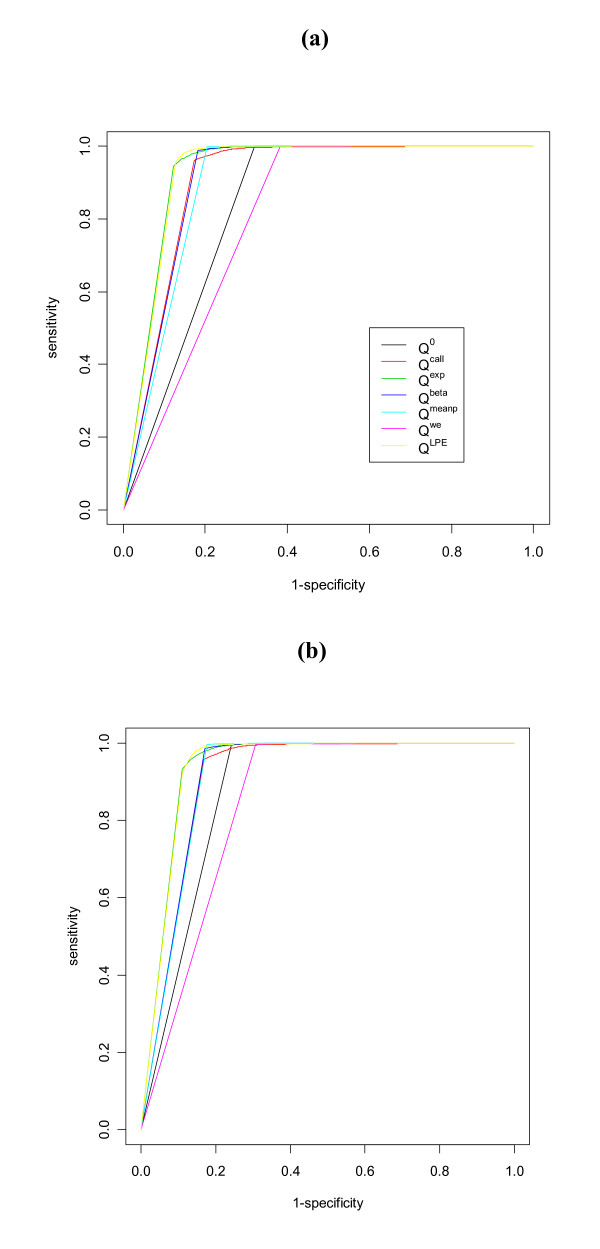
Receiver Operator Characteristic (ROC) plots for tests of differential expression in the simulated data with treatment effect *δg *= 2.0. Six weighted tests and the local pooled error (LPE) test are compared. (a) p-values unadjusted for multiple testing, (b) p-values adjusted by the weighted Benjamini and Hochberg (WBH) multiple testing method.

**Figure 2:**
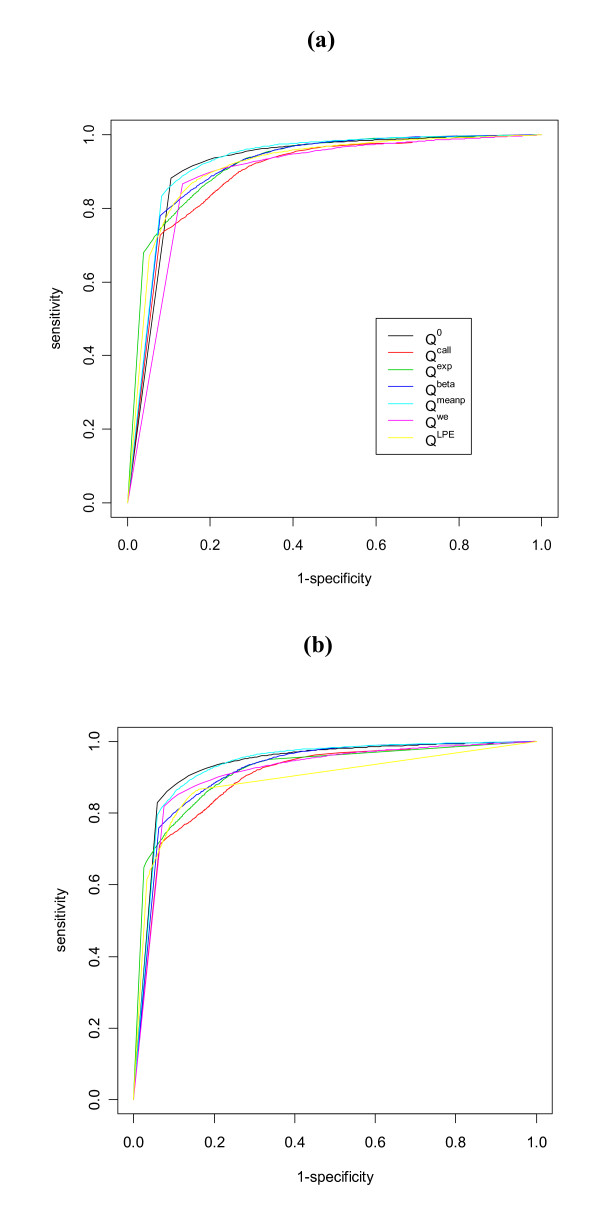
Receiver Operator Characteristic (ROC) plots for tests of differential expression in the simulated data with treatment effect *δg *= 1.0. (a) p-values unadjusted for multiple testing, (b) p-values adjusted by the weighted Benjamini and Hochberg (WBH) multiple testing method.

The performance of our proposed methods was evaluated using simulated probe level data generated from a model incorporating probe level effects, optical noise, and non-specific binding, as well as true signals [14]. We have run five simulation models following the simulation procedures described in Materials and Methods. Treatment effects on the signal were varied between 0.5 (very small) and 2.5 (very large) in the five models. To compare performance, we used Summarized Receiver Operating Characteristic (SROC) curves, where the test sensitivities and specificities (true positive and true negative proportions) for a range of p-value cutoffs (or FDR cutoffs for results with multiple testing adjustments) were averaged over 100 simulated datasets. Figures 1 and 2 show SROC curves of models 2 and 4, where large and small treatment effect sizes, respectively, were chosen in the generated models.

The SROC curve's overall behavior can be measured by the Area Under the Curve (AUC) [19]. Table 2 shows AUCs for five simulation models under different weighting and multiple testing strategies. As seen from the table and figures, weighted t-statistics based on quality measure Qgcall
 MathType@MTEF@5@5@+=feaafiart1ev1aaatCvAUfKttLearuWrP9MDH5MBPbIqV92AaeXatLxBI9gBaebbnrfifHhDYfgasaacH8akY=wiFfYdH8Gipec8Eeeu0xXdbba9frFj0=OqFfea0dXdd9vqai=hGuQ8kuc9pgc9s8qqaq=dirpe0xb9q8qiLsFr0=vr0=vr0dc8meaabaqaciaacaGaaeqabaqabeGadaaakeaacqWGrbqudaqhaaWcbaGaem4zaCgabaGaem4yamMaemyyaeMaemiBaWMaemiBaWgaaaaa@34B7@, in which a gene was excluded if one or more arrays have an absent call, had the lowest AUC in all models. Whether p-values were unadjusted or adjusted by the weighted Benjamini-Hochberg (WBH) method, Qgexp⁡
 MathType@MTEF@5@5@+=feaafiart1ev1aaatCvAUfKttLearuWrP9MDH5MBPbIqV92AaeXatLxBI9gBaebbnrfifHhDYfgasaacH8akY=wiFfYdH8Gipec8Eeeu0xXdbba9frFj0=OqFfea0dXdd9vqai=hGuQ8kuc9pgc9s8qqaq=dirpe0xb9q8qiLsFr0=vr0=vr0dc8meaabaqaciaacaGaaeqabaqabeGadaaakeaacqWGrbqudaqhaaWcbaGaem4zaCgabaGagiyzauMaeiiEaGNaeiiCaahaaaaa@338F@ outperformed the other quality scores in models 1, 2, and 3, where a moderate to large treatment effect was chosen. Although Qgmeanp
 MathType@MTEF@5@5@+=feaafiart1ev1aaatCvAUfKttLearuWrP9MDH5MBPbIqV92AaeXatLxBI9gBaebbnrfifHhDYfgasaacH8akY=wiFfYdH8Gipec8Eeeu0xXdbba9frFj0=OqFfea0dXdd9vqai=hGuQ8kuc9pgc9s8qqaq=dirpe0xb9q8qiLsFr0=vr0=vr0dc8meaabaqaciaacaGaaeqabaqabeGadaaakeaacqWGrbqudaqhaaWcbaGaem4zaCgabaGaemyBa0MaemyzauMaemyyaeMaemOBa4MaemiCaahaaaaa@362A@ had the overall best performance for models 4 and 5, where a small treatment effect was used, Qgexp⁡
 MathType@MTEF@5@5@+=feaafiart1ev1aaatCvAUfKttLearuWrP9MDH5MBPbIqV92AaeXatLxBI9gBaebbnrfifHhDYfgasaacH8akY=wiFfYdH8Gipec8Eeeu0xXdbba9frFj0=OqFfea0dXdd9vqai=hGuQ8kuc9pgc9s8qqaq=dirpe0xb9q8qiLsFr0=vr0=vr0dc8meaabaqaciaacaGaaeqabaqabeGadaaakeaacqWGrbqudaqhaaWcbaGaem4zaCgabaGagiyzauMaeiiEaGNaeiiCaahaaaaa@338F@ has the best sensitivity when specificity is larger than 95% (shown in Figure 2). We also observed that Qgexp⁡
 MathType@MTEF@5@5@+=feaafiart1ev1aaatCvAUfKttLearuWrP9MDH5MBPbIqV92AaeXatLxBI9gBaebbnrfifHhDYfgasaacH8akY=wiFfYdH8Gipec8Eeeu0xXdbba9frFj0=OqFfea0dXdd9vqai=hGuQ8kuc9pgc9s8qqaq=dirpe0xb9q8qiLsFr0=vr0=vr0dc8meaabaqaciaacaGaaeqabaqabeGadaaakeaacqWGrbqudaqhaaWcbaGaem4zaCgabaGagiyzauMaeiiEaGNaeiiCaahaaaaa@338F@ had similar performance to QgLPE
 MathType@MTEF@5@5@+=feaafiart1ev1aaatCvAUfKttLearuWrP9MDH5MBPbIqV92AaeXatLxBI9gBaebbnrfifHhDYfgasaacH8akY=wiFfYdH8Gipec8Eeeu0xXdbba9frFj0=OqFfea0dXdd9vqai=hGuQ8kuc9pgc9s8qqaq=dirpe0xb9q8qiLsFr0=vr0=vr0dc8meaabaqaciaacaGaaeqabaqabeGadaaakeaacqWGrbqudaqhaaWcbaGaem4zaCgabaGaemitaWKaemiuaaLaemyraueaaaaa@32B8@ for models 1, 2 and 3, but Qgexp⁡
 MathType@MTEF@5@5@+=feaafiart1ev1aaatCvAUfKttLearuWrP9MDH5MBPbIqV92AaeXatLxBI9gBaebbnrfifHhDYfgasaacH8akY=wiFfYdH8Gipec8Eeeu0xXdbba9frFj0=OqFfea0dXdd9vqai=hGuQ8kuc9pgc9s8qqaq=dirpe0xb9q8qiLsFr0=vr0=vr0dc8meaabaqaciaacaGaaeqabaqabeGadaaakeaacqWGrbqudaqhaaWcbaGaem4zaCgabaGagiyzauMaeiiEaGNaeiiCaahaaaaa@338F@ outperformed QgLPE
 MathType@MTEF@5@5@+=feaafiart1ev1aaatCvAUfKttLearuWrP9MDH5MBPbIqV92AaeXatLxBI9gBaebbnrfifHhDYfgasaacH8akY=wiFfYdH8Gipec8Eeeu0xXdbba9frFj0=OqFfea0dXdd9vqai=hGuQ8kuc9pgc9s8qqaq=dirpe0xb9q8qiLsFr0=vr0=vr0dc8meaabaqaciaacaGaaeqabaqabeGadaaakeaacqWGrbqudaqhaaWcbaGaem4zaCgabaGaemitaWKaemiuaaLaemyraueaaaaa@32B8@ for models 4 and 5. Qgbeta
 MathType@MTEF@5@5@+=feaafiart1ev1aaatCvAUfKttLearuWrP9MDH5MBPbIqV92AaeXatLxBI9gBaebbnrfifHhDYfgasaacH8akY=wiFfYdH8Gipec8Eeeu0xXdbba9frFj0=OqFfea0dXdd9vqai=hGuQ8kuc9pgc9s8qqaq=dirpe0xb9q8qiLsFr0=vr0=vr0dc8meaabaqaciaacaGaaeqabaqabeGadaaakeaacqWGrbqudaqhaaWcbaGaem4zaCgabaGaemOyaiMaemyzauMaemiDaqNaemyyaegaaaaa@34B7@ has slightly worse performance than Qgexp⁡
 MathType@MTEF@5@5@+=feaafiart1ev1aaatCvAUfKttLearuWrP9MDH5MBPbIqV92AaeXatLxBI9gBaebbnrfifHhDYfgasaacH8akY=wiFfYdH8Gipec8Eeeu0xXdbba9frFj0=OqFfea0dXdd9vqai=hGuQ8kuc9pgc9s8qqaq=dirpe0xb9q8qiLsFr0=vr0=vr0dc8meaabaqaciaacaGaaeqabaqabeGadaaakeaacqWGrbqudaqhaaWcbaGaem4zaCgabaGagiyzauMaeiiEaGNaeiiCaahaaaaa@338F@ for models 1,2 and 3, but their performance is almost the same for models 4 and 5; conversely, the statistic Qgwe
 MathType@MTEF@5@5@+=feaafiart1ev1aaatCvAUfKttLearuWrP9MDH5MBPbIqV92AaeXatLxBI9gBaebbnrfifHhDYfgasaacH8akY=wiFfYdH8Gipec8Eeeu0xXdbba9frFj0=OqFfea0dXdd9vqai=hGuQ8kuc9pgc9s8qqaq=dirpe0xb9q8qiLsFr0=vr0=vr0dc8meaabaqaciaacaGaaeqabaqabeGadaaakeaacqWGrbqudaqhaaWcbaGaem4zaCgabaGaem4DaCNaemyzaugaaaaa@3225@ performed better, relative to the other methods, for small effect sizes. The performance of no filtering at all, Qg0
 MathType@MTEF@5@5@+=feaafiart1ev1aaatCvAUfKttLearuWrP9MDH5MBPbIqV92AaeXatLxBI9gBaebbnrfifHhDYfgasaacH8akY=wiFfYdH8Gipec8Eeeu0xXdbba9frFj0=OqFfea0dXdd9vqai=hGuQ8kuc9pgc9s8qqaq=dirpe0xb9q8qiLsFr0=vr0=vr0dc8meaabaqaciaacaGaaeqabaqabeGadaaakeaacqWGrbqudaqhaaWcbaGaem4zaCgabaGaeGimaadaaaaa@3049@, was also a good choice for small changes in gene expression. Therefore, the best choice of weighting statistic in the presence of adjustments for multiple testing appears to depend on the size of the treatment effects. We also found that it is better to define quality measures directly from Affymetrix detection p-values rather than from the Affymetrix Present/Marginal/Absent calls.

### Duchenne muscular dystrophy vs. normal muscle

Haslett et al. [16] compared gene expression between 12 quadriceps biopsies from Duchenne muscular dystrophy (DMD) patients and 12 quadriceps biopsies of normal skeletal muscle. Using a method called geometric fold change (GFC), they identified 133 differentially expressed genes (139 probesets) with a permutation-based false discovery rate of 2.3 × 10^-3^; Of these, 12 genes (13 probesets) were confirmed by RT-PCR. This set will be referred to as the "RT-PCR" probesets.

Tables 3 and 4 compare the agreement between probesets selected by our test statistics and Haslett et al. [16]. In Table 3, for each test statistic, we selected the top T = 30, 50, 100 or 139 significantly expressed probesets, ranked based on our adjusted false discovery rates, and we counted the number of these probesets that were also in the top T probesets identified by GFC and ranked based on their absolute fold changes. Table 4 shows a similar comparison for the RT-PCR set.

The measure Qgcall
 MathType@MTEF@5@5@+=feaafiart1ev1aaatCvAUfKttLearuWrP9MDH5MBPbIqV92AaeXatLxBI9gBaebbnrfifHhDYfgasaacH8akY=wiFfYdH8Gipec8Eeeu0xXdbba9frFj0=OqFfea0dXdd9vqai=hGuQ8kuc9pgc9s8qqaq=dirpe0xb9q8qiLsFr0=vr0=vr0dc8meaabaqaciaacaGaaeqabaqabeGadaaakeaacqWGrbqudaqhaaWcbaGaem4zaCgabaGaem4yamMaemyyaeMaemiBaWMaemiBaWgaaaaa@34B7@ had the worst performance in concordance for the lists of top T probesets in Table 3, and for identifying RT-PCR probesets in Table 4. The weighted t-statistics based on Qgexp⁡
 MathType@MTEF@5@5@+=feaafiart1ev1aaatCvAUfKttLearuWrP9MDH5MBPbIqV92AaeXatLxBI9gBaebbnrfifHhDYfgasaacH8akY=wiFfYdH8Gipec8Eeeu0xXdbba9frFj0=OqFfea0dXdd9vqai=hGuQ8kuc9pgc9s8qqaq=dirpe0xb9q8qiLsFr0=vr0=vr0dc8meaabaqaciaacaGaaeqabaqabeGadaaakeaacqWGrbqudaqhaaWcbaGaem4zaCgabaGagiyzauMaeiiEaGNaeiiCaahaaaaa@338F@ and Qgbeta
 MathType@MTEF@5@5@+=feaafiart1ev1aaatCvAUfKttLearuWrP9MDH5MBPbIqV92AaeXatLxBI9gBaebbnrfifHhDYfgasaacH8akY=wiFfYdH8Gipec8Eeeu0xXdbba9frFj0=OqFfea0dXdd9vqai=hGuQ8kuc9pgc9s8qqaq=dirpe0xb9q8qiLsFr0=vr0=vr0dc8meaabaqaciaacaGaaeqabaqabeGadaaakeaacqWGrbqudaqhaaWcbaGaem4zaCgabaGaemOyaiMaemyzauMaemiDaqNaemyyaegaaaaa@34B7@ performed quite similarly, and gave results that were close also to three other statistics (Qg0
 MathType@MTEF@5@5@+=feaafiart1ev1aaatCvAUfKttLearuWrP9MDH5MBPbIqV92AaeXatLxBI9gBaebbnrfifHhDYfgasaacH8akY=wiFfYdH8Gipec8Eeeu0xXdbba9frFj0=OqFfea0dXdd9vqai=hGuQ8kuc9pgc9s8qqaq=dirpe0xb9q8qiLsFr0=vr0=vr0dc8meaabaqaciaacaGaaeqabaqabeGadaaakeaacqWGrbqudaqhaaWcbaGaem4zaCgabaGaeGimaadaaaaa@3049@Qgmeanp
 MathType@MTEF@5@5@+=feaafiart1ev1aaatCvAUfKttLearuWrP9MDH5MBPbIqV92AaeXatLxBI9gBaebbnrfifHhDYfgasaacH8akY=wiFfYdH8Gipec8Eeeu0xXdbba9frFj0=OqFfea0dXdd9vqai=hGuQ8kuc9pgc9s8qqaq=dirpe0xb9q8qiLsFr0=vr0=vr0dc8meaabaqaciaacaGaaeqabaqabeGadaaakeaacqWGrbqudaqhaaWcbaGaem4zaCgabaGaemyBa0MaemyzauMaemyyaeMaemOBa4MaemiCaahaaaaa@362A@Qgwe
 MathType@MTEF@5@5@+=feaafiart1ev1aaatCvAUfKttLearuWrP9MDH5MBPbIqV92AaeXatLxBI9gBaebbnrfifHhDYfgasaacH8akY=wiFfYdH8Gipec8Eeeu0xXdbba9frFj0=OqFfea0dXdd9vqai=hGuQ8kuc9pgc9s8qqaq=dirpe0xb9q8qiLsFr0=vr0=vr0dc8meaabaqaciaacaGaaeqabaqabeGadaaakeaacqWGrbqudaqhaaWcbaGaem4zaCgabaGaem4DaCNaemyzaugaaaaa@3225@), but Qgexp⁡
 MathType@MTEF@5@5@+=feaafiart1ev1aaatCvAUfKttLearuWrP9MDH5MBPbIqV92AaeXatLxBI9gBaebbnrfifHhDYfgasaacH8akY=wiFfYdH8Gipec8Eeeu0xXdbba9frFj0=OqFfea0dXdd9vqai=hGuQ8kuc9pgc9s8qqaq=dirpe0xb9q8qiLsFr0=vr0=vr0dc8meaabaqaciaacaGaaeqabaqabeGadaaakeaacqWGrbqudaqhaaWcbaGaem4zaCgabaGagiyzauMaeiiEaGNaeiiCaahaaaaa@338F@ showed slightly better results in Table 3 than these other methods. Interestingly, the FDR values in Table 3 associated with Qgexp⁡
 MathType@MTEF@5@5@+=feaafiart1ev1aaatCvAUfKttLearuWrP9MDH5MBPbIqV92AaeXatLxBI9gBaebbnrfifHhDYfgasaacH8akY=wiFfYdH8Gipec8Eeeu0xXdbba9frFj0=OqFfea0dXdd9vqai=hGuQ8kuc9pgc9s8qqaq=dirpe0xb9q8qiLsFr0=vr0=vr0dc8meaabaqaciaacaGaaeqabaqabeGadaaakeaacqWGrbqudaqhaaWcbaGaem4zaCgabaGagiyzauMaeiiEaGNaeiiCaahaaaaa@338F@ are visibly larger than for the other methods. Although there is more agreement between GFC and QgLPE
 MathType@MTEF@5@5@+=feaafiart1ev1aaatCvAUfKttLearuWrP9MDH5MBPbIqV92AaeXatLxBI9gBaebbnrfifHhDYfgasaacH8akY=wiFfYdH8Gipec8Eeeu0xXdbba9frFj0=OqFfea0dXdd9vqai=hGuQ8kuc9pgc9s8qqaq=dirpe0xb9q8qiLsFr0=vr0=vr0dc8meaabaqaciaacaGaaeqabaqabeGadaaakeaacqWGrbqudaqhaaWcbaGaem4zaCgabaGaemitaWKaemiuaaLaemyraueaaaaa@32B8@ in Table 3 than for other methods when the number of selected probesets is small (< = 100), the agreement decreases when more probesets are selected, even though the FDR estimates are smaller. This statistic is obviously very different from the others. When examining Table 3 for T = 30, agreement for all methods with the GFC paper was slightly better using MAS5. This may not be surprising since Haslett et al. [16] used MAS5 also, although, in Table 3, this relationship not entirely consistent across different values of T. In Table 4, however, the agreement is always better using MAS5.

### Analysis of Choe's spiked-in data

The availability of data from spiked-in experiments (Choe et al. [17]) provides an excellent opportunity to examine the performance of our weighted statistics on real data where the answers are known. Selected transcripts were added at a range of known concentrations; some were chosen to have differential expression between two groups of samples (true positive changes in expression); others were spiked-in at the same concentration in the two groups (true negative changes in expression).

**Figure 3:**
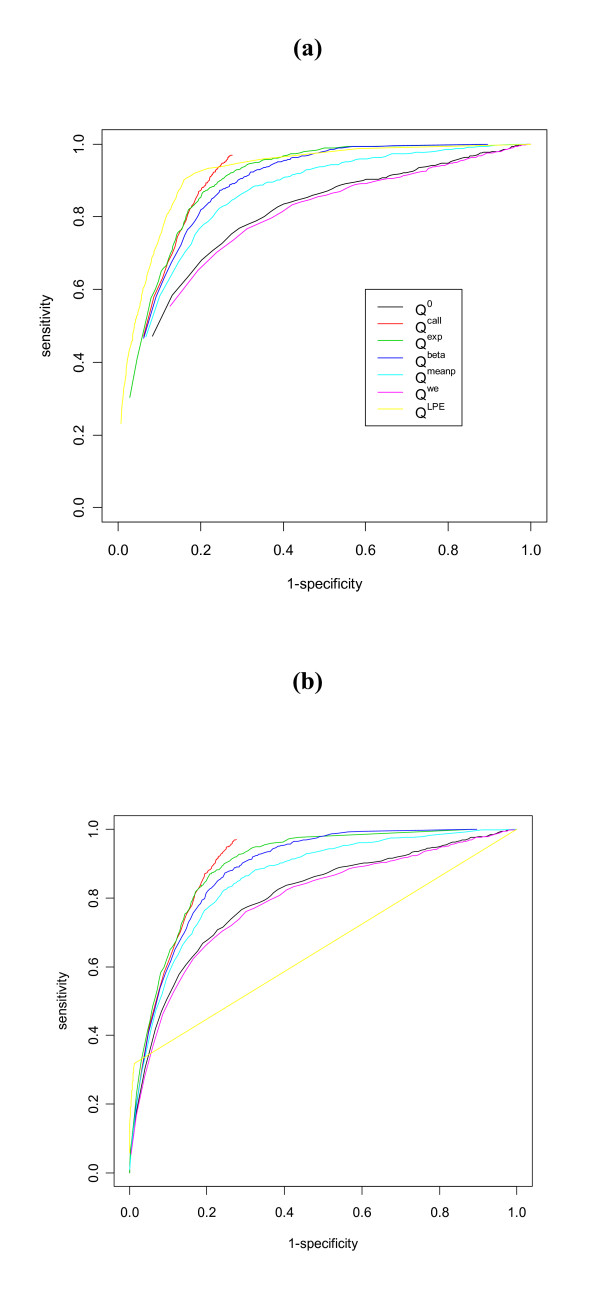
Receiver Operator Characteristic (ROC) plots for tests of differential expression in Choe's spiked-in data summarized by MAS5. (a) p-values unadjusted for multiple testing, (b) p-values adjusted by the weighted Benjamini and Hochberg (WBH) multiple testing method.

**Figure 4:**
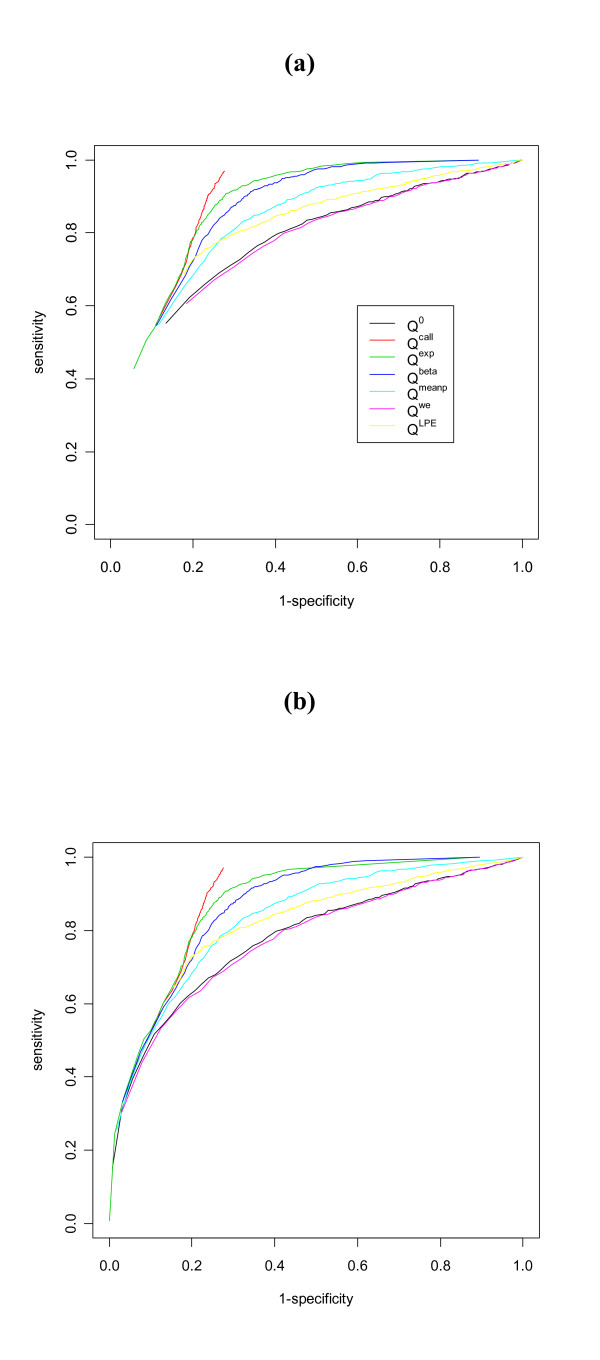
Receiver Operator Characteristic (ROC) plots for tests of differential expression in Choe's spiked-in data was summarized by RMA. (a) p-values unadjusted for multiple testing, (b) p-values adjusted by the weighted Benjamini and Hochberg (WBH) multiple testing method.

Figures 3 and 4 show the ROC curves for the comparison of the two groups for MAS5 and RMA, respectively. Table 5 shows the AUC under different weighting and multiple testing strategies. For both the RMA and MAS5 strategies, it is still clear that whether p-values were unadjusted or adjusted by the WBH method, Qgexp⁡
 MathType@MTEF@5@5@+=feaafiart1ev1aaatCvAUfKttLearuWrP9MDH5MBPbIqV92AaeXatLxBI9gBaebbnrfifHhDYfgasaacH8akY=wiFfYdH8Gipec8Eeeu0xXdbba9frFj0=OqFfea0dXdd9vqai=hGuQ8kuc9pgc9s8qqaq=dirpe0xb9q8qiLsFr0=vr0=vr0dc8meaabaqaciaacaGaaeqabaqabeGadaaakeaacqWGrbqudaqhaaWcbaGaem4zaCgabaGagiyzauMaeiiEaGNaeiiCaahaaaaa@338F@ outperforms the other quality scores. However, unlike the results in the DMD as well as the simulated data, here the quality measure Qgcall
 MathType@MTEF@5@5@+=feaafiart1ev1aaatCvAUfKttLearuWrP9MDH5MBPbIqV92AaeXatLxBI9gBaebbnrfifHhDYfgasaacH8akY=wiFfYdH8Gipec8Eeeu0xXdbba9frFj0=OqFfea0dXdd9vqai=hGuQ8kuc9pgc9s8qqaq=dirpe0xb9q8qiLsFr0=vr0=vr0dc8meaabaqaciaacaGaaeqabaqabeGadaaakeaacqWGrbqudaqhaaWcbaGaem4zaCgabaGaem4yamMaemyyaeMaemiBaWMaemiBaWgaaaaa@34B7@ had better performance than Qg0
 MathType@MTEF@5@5@+=feaafiart1ev1aaatCvAUfKttLearuWrP9MDH5MBPbIqV92AaeXatLxBI9gBaebbnrfifHhDYfgasaacH8akY=wiFfYdH8Gipec8Eeeu0xXdbba9frFj0=OqFfea0dXdd9vqai=hGuQ8kuc9pgc9s8qqaq=dirpe0xb9q8qiLsFr0=vr0=vr0dc8meaabaqaciaacaGaaeqabaqabeGadaaakeaacqWGrbqudaqhaaWcbaGaem4zaCgabaGaeGimaadaaaaa@3049@, Qgmeanp
 MathType@MTEF@5@5@+=feaafiart1ev1aaatCvAUfKttLearuWrP9MDH5MBPbIqV92AaeXatLxBI9gBaebbnrfifHhDYfgasaacH8akY=wiFfYdH8Gipec8Eeeu0xXdbba9frFj0=OqFfea0dXdd9vqai=hGuQ8kuc9pgc9s8qqaq=dirpe0xb9q8qiLsFr0=vr0=vr0dc8meaabaqaciaacaGaaeqabaqabeGadaaakeaacqWGrbqudaqhaaWcbaGaem4zaCgabaGaemyBa0MaemyzauMaemyyaeMaemOBa4MaemiCaahaaaaa@362A@ and Qgwe
 MathType@MTEF@5@5@+=feaafiart1ev1aaatCvAUfKttLearuWrP9MDH5MBPbIqV92AaeXatLxBI9gBaebbnrfifHhDYfgasaacH8akY=wiFfYdH8Gipec8Eeeu0xXdbba9frFj0=OqFfea0dXdd9vqai=hGuQ8kuc9pgc9s8qqaq=dirpe0xb9q8qiLsFr0=vr0=vr0dc8meaabaqaciaacaGaaeqabaqabeGadaaakeaacqWGrbqudaqhaaWcbaGaem4zaCgabaGaem4DaCNaemyzaugaaaaa@3225@. In general, the results based on the MAS5 strategy are slightly better than those based on RMA for any quality-based test statistic, but the relative rankings of the different statistics remain almost the same across the two summarization methods. The LPE test appears to be particularly sensitive to the summarization method. It performs very well for the MAS5 method without multiple testing corrections and very poorly when the BH multiple testing is used. Test statistics based on Qgexp⁡
 MathType@MTEF@5@5@+=feaafiart1ev1aaatCvAUfKttLearuWrP9MDH5MBPbIqV92AaeXatLxBI9gBaebbnrfifHhDYfgasaacH8akY=wiFfYdH8Gipec8Eeeu0xXdbba9frFj0=OqFfea0dXdd9vqai=hGuQ8kuc9pgc9s8qqaq=dirpe0xb9q8qiLsFr0=vr0=vr0dc8meaabaqaciaacaGaaeqabaqabeGadaaakeaacqWGrbqudaqhaaWcbaGaem4zaCgabaGagiyzauMaeiiEaGNaeiiCaahaaaaa@338F@ and Qgbeta
 MathType@MTEF@5@5@+=feaafiart1ev1aaatCvAUfKttLearuWrP9MDH5MBPbIqV92AaeXatLxBI9gBaebbnrfifHhDYfgasaacH8akY=wiFfYdH8Gipec8Eeeu0xXdbba9frFj0=OqFfea0dXdd9vqai=hGuQ8kuc9pgc9s8qqaq=dirpe0xb9q8qiLsFr0=vr0=vr0dc8meaabaqaciaacaGaaeqabaqabeGadaaakeaacqWGrbqudaqhaaWcbaGaem4zaCgabaGaemOyaiMaemyzauMaemiDaqNaemyyaegaaaaa@34B7@ had better performance than the LPE test when p-values were adjusted by the WBH in both MAS5 and RMA.

## Discussion

In traditional gene selection methods, pre-filtering and selection are two separate procedures, and commonly used pre-filtering methods are based on Affymetrix calls [6-9]. We unified gene filtering and gene selection procedures together, where the importance of a gene is measured by its quality score, defined across all arrays and experimental groups, rather than by a given cutoff call value. The methods, therefore, overcome the shortcomings of the traditional methods to filter out genes before any high-level data analysis, such as gene selection, is carried out.

Our measure Qgcall
 MathType@MTEF@5@5@+=feaafiart1ev1aaatCvAUfKttLearuWrP9MDH5MBPbIqV92AaeXatLxBI9gBaebbnrfifHhDYfgasaacH8akY=wiFfYdH8Gipec8Eeeu0xXdbba9frFj0=OqFfea0dXdd9vqai=hGuQ8kuc9pgc9s8qqaq=dirpe0xb9q8qiLsFr0=vr0=vr0dc8meaabaqaciaacaGaaeqabaqabeGadaaakeaacqWGrbqudaqhaaWcbaGaem4zaCgabaGaem4yamMaemyyaeMaemiBaWMaemiBaWgaaaaa@34B7@ is essentially the same as the traditional method of keeping only probesets where all arrays record a present call. Our results clearly demonstrated that the weighted t-statistic based on Qgcall
 MathType@MTEF@5@5@+=feaafiart1ev1aaatCvAUfKttLearuWrP9MDH5MBPbIqV92AaeXatLxBI9gBaebbnrfifHhDYfgasaacH8akY=wiFfYdH8Gipec8Eeeu0xXdbba9frFj0=OqFfea0dXdd9vqai=hGuQ8kuc9pgc9s8qqaq=dirpe0xb9q8qiLsFr0=vr0=vr0dc8meaabaqaciaacaGaaeqabaqabeGadaaakeaacqWGrbqudaqhaaWcbaGaem4zaCgabaGaem4yamMaemyyaeMaemiBaWMaemiBaWgaaaaa@34B7@ has the worst performance in the real data and all simulation models; Pounds and Cheng [11] also found that simple filtering based on Present/Marginal/Absent calls was a poor choice. However, this statistic performed well in the spiked-in data. This artificially-created dataset contained only transcripts that were added in known concentrations, therefore the expression signals are likely to be much more consistent across arrays than signals from different individuals.

The best choice of weighting statistic in the presence of adjustments for multiple testing appears to depend on the size of the treatment effects. The exponential model measure, Qgexp⁡
 MathType@MTEF@5@5@+=feaafiart1ev1aaatCvAUfKttLearuWrP9MDH5MBPbIqV92AaeXatLxBI9gBaebbnrfifHhDYfgasaacH8akY=wiFfYdH8Gipec8Eeeu0xXdbba9frFj0=OqFfea0dXdd9vqai=hGuQ8kuc9pgc9s8qqaq=dirpe0xb9q8qiLsFr0=vr0=vr0dc8meaabaqaciaacaGaaeqabaqabeGadaaakeaacqWGrbqudaqhaaWcbaGaem4zaCgabaGagiyzauMaeiiEaGNaeiiCaahaaaaa@338F@, appears to have a slight advantage over the one-parameter beta distribution Qgbeta
 MathType@MTEF@5@5@+=feaafiart1ev1aaatCvAUfKttLearuWrP9MDH5MBPbIqV92AaeXatLxBI9gBaebbnrfifHhDYfgasaacH8akY=wiFfYdH8Gipec8Eeeu0xXdbba9frFj0=OqFfea0dXdd9vqai=hGuQ8kuc9pgc9s8qqaq=dirpe0xb9q8qiLsFr0=vr0=vr0dc8meaabaqaciaacaGaaeqabaqabeGadaaakeaacqWGrbqudaqhaaWcbaGaem4zaCgabaGaemOyaiMaemyzauMaemiDaqNaemyyaegaaaaa@34B7@, although for small effect sizes they perform very similarly. The distribution of quality scores for the exponential model gives, in general, lower weight to probesets with small detection p-values than the beta distribution. This may lead to a small reduction in sensitivity – the effect of these lower weights can be seen when examining the FDR cutoffs in the DMD data, and in the figures. However, these lower weights also improve specificity which tends to lead to better overall prediction.

For quality measures Qgexp⁡
 MathType@MTEF@5@5@+=feaafiart1ev1aaatCvAUfKttLearuWrP9MDH5MBPbIqV92AaeXatLxBI9gBaebbnrfifHhDYfgasaacH8akY=wiFfYdH8Gipec8Eeeu0xXdbba9frFj0=OqFfea0dXdd9vqai=hGuQ8kuc9pgc9s8qqaq=dirpe0xb9q8qiLsFr0=vr0=vr0dc8meaabaqaciaacaGaaeqabaqabeGadaaakeaacqWGrbqudaqhaaWcbaGaem4zaCgabaGagiyzauMaeiiEaGNaeiiCaahaaaaa@338F@ and Qgbeta
 MathType@MTEF@5@5@+=feaafiart1ev1aaatCvAUfKttLearuWrP9MDH5MBPbIqV92AaeXatLxBI9gBaebbnrfifHhDYfgasaacH8akY=wiFfYdH8Gipec8Eeeu0xXdbba9frFj0=OqFfea0dXdd9vqai=hGuQ8kuc9pgc9s8qqaq=dirpe0xb9q8qiLsFr0=vr0=vr0dc8meaabaqaciaacaGaaeqabaqabeGadaaakeaacqWGrbqudaqhaaWcbaGaem4zaCgabaGaemOyaiMaemyzauMaemiDaqNaemyyaegaaaaa@34B7@, the performance of the weight depends on the sensitivity parameter *ν*. In order to analyze the effect of different values of parameter *ν *on our results, we did another simulation study. Table 6 summarizes the sensitivity and specificity of the proposed test procedures to detect differentially expressed genes (using the weighted Benjamini-Hochberg procedure to control the false discovery rate at 5%), as a function of *ν *when the treatment effect was set to 2.0. The sensitivity decreases and specificity increases as *ν *gets smaller. The sensitivity associated with Qgbeta
 MathType@MTEF@5@5@+=feaafiart1ev1aaatCvAUfKttLearuWrP9MDH5MBPbIqV92AaeXatLxBI9gBaebbnrfifHhDYfgasaacH8akY=wiFfYdH8Gipec8Eeeu0xXdbba9frFj0=OqFfea0dXdd9vqai=hGuQ8kuc9pgc9s8qqaq=dirpe0xb9q8qiLsFr0=vr0=vr0dc8meaabaqaciaacaGaaeqabaqabeGadaaakeaacqWGrbqudaqhaaWcbaGaem4zaCgabaGaemOyaiMaemyzauMaemiDaqNaemyyaegaaaaa@34B7@ is slightly better than Qgexp⁡
 MathType@MTEF@5@5@+=feaafiart1ev1aaatCvAUfKttLearuWrP9MDH5MBPbIqV92AaeXatLxBI9gBaebbnrfifHhDYfgasaacH8akY=wiFfYdH8Gipec8Eeeu0xXdbba9frFj0=OqFfea0dXdd9vqai=hGuQ8kuc9pgc9s8qqaq=dirpe0xb9q8qiLsFr0=vr0=vr0dc8meaabaqaciaacaGaaeqabaqabeGadaaakeaacqWGrbqudaqhaaWcbaGaem4zaCgabaGagiyzauMaeiiEaGNaeiiCaahaaaaa@338F@ across this table, but the specificity is worse. It can be argued that the optimal choice of *ν *is the one that comes closest to perfect prediction. For *ν *= 0.05 and 0.01, the Euclidean distances from the point where sensitivity = 1 and specificity = 1 are 0.153 and 0.157, for the exponential distribution, and 0.219 and 0.221, for the beta distribution, respectively. Therefore, we fixed *ν *= 0.05 in our analysis; this choice can be thought of as a p-value of 0.05, leading to the interpretation that a representative transcript from a particular experimental group will be called present when the expected (under the assumed distribution) p-value <0.05. In fact, the significance level of 0.05 is widely used in statistical hypothesis testing and is comparable to the thresholds for Present/Marginal/Absent calls used in the Affymetrix software.

It is now a common practice to use procedures for controlling multiple testing when identifying differentially expressed genes. Various procedures, such as the Bonferroni correction, the Benjamini and Hochberg false discovery rate [4], or the Benjamini and Yekutieli false discovery rate [20], have been widely used. Due to the quality adjustment in our proposed t-statistics, we can no longer assume that the p-values *p*_*tg *_have a uniform distribution under the null hypothesis, and we can not assume that the test statistics have identical distributions. Therefore, we developed weighted multiple testing procedures. Our findings showed that the proposed weighted Benjamini and Hochberg (WBH) adjustment procedure is better than the weighted Bonferroni (WB) and weighted Benjamini and Yekutieli (WBY) adjustment procedures (results not shown). However, we also observed that the proposed t-statistics using WB and WBY had poor sensitivity, regardless of the type of quality measure score (data not shown). We are planning to further extend weighted multiple testing methods in order to redefine Storey and Tibshirani's positive false discovery rate (pFDR) [21], where the test statistics are assumed to be identically distributed.

Several other modified t-statistics, such as SAM [22], penalized t-statistics [23], or the local pooled error method used here [18], have been developed for microarray data analysis. All of these methods focus on overcoming the shortcoming of the ordinary t-statistics for ranking genes, due to unstable variance estimates that may arise when sample size is small. Each method used a slightly different strategy to estimate a penalty parameter for smoothing unstable variance estimates, using information from all genes rather than relying solely on variance estimates from an individual gene. However, as we discussed in the "Background" Section, the quality of this information is not the same across genes. Therefore, the estimate of the penalty parameter may not be reliable if we assume that all gene-specific information has the same quality. Here, we take another strategy to improve the performance of ordinary t statistics by putting a high weight for the genes with high quality and a low weight for those with low quality in the t-statistic calculations. The initial comparison of our strategy with the LPE test demonstrated that our weighted tests based on Qgexp⁡
 MathType@MTEF@5@5@+=feaafiart1ev1aaatCvAUfKttLearuWrP9MDH5MBPbIqV92AaeXatLxBI9gBaebbnrfifHhDYfgasaacH8akY=wiFfYdH8Gipec8Eeeu0xXdbba9frFj0=OqFfea0dXdd9vqai=hGuQ8kuc9pgc9s8qqaq=dirpe0xb9q8qiLsFr0=vr0=vr0dc8meaabaqaciaacaGaaeqabaqabeGadaaakeaacqWGrbqudaqhaaWcbaGaem4zaCgabaGagiyzauMaeiiEaGNaeiiCaahaaaaa@338F@ and Qgbeta
 MathType@MTEF@5@5@+=feaafiart1ev1aaatCvAUfKttLearuWrP9MDH5MBPbIqV92AaeXatLxBI9gBaebbnrfifHhDYfgasaacH8akY=wiFfYdH8Gipec8Eeeu0xXdbba9frFj0=OqFfea0dXdd9vqai=hGuQ8kuc9pgc9s8qqaq=dirpe0xb9q8qiLsFr0=vr0=vr0dc8meaabaqaciaacaGaaeqabaqabeGadaaakeaacqWGrbqudaqhaaWcbaGaem4zaCgabaGaemOyaiMaemyzauMaemiDaqNaemyyaegaaaaa@34B7@ are promising – AUC results were fairly similar, and better in several cases. Although our weighting strategy could be combined with a revised variance-smoothing algorithm to potentially improve the performance of the test statistics in small samples, this was not the focus of this work.

It should be noted that the probe-level data analysis (such as background correction, normalization and summarization methods) may influence the results of the test procedures we discussed. Our initial analysis of real data and spiked-in data show that the weighted test statistics based on the MAS5 strategy may have slightly better performance than those based on the RMA strategy. In future investigations, we plan to explore how different probe-level data analysis methods, such as MAS5, dCHIP, RMA, PLIER and gcRMA, and different detection p-value calculation methods [17], may influence our weighting strategy in detail.

Our results showed that some true differences are missed with any filtering method, as was noted by others [11]. It is known that the mismatch probes measure some true signal [12], and therefore is possible that, especially for genes expressed at a low level, quality scores for real data are lower than in our simulated data. Defining an alternative to the detection p-value that does not depend on the mismatch data may lead to better sensitivity. However, if mismatch probes do contain signal, and a study wishes to identify small changes in gene expression, the decision to use any filter at all must be carefully considered, given that some true effects are likely to be excluded. In addition, when effects are small, it must be realized that using multiple testing corrections will also lead to the exclusion of real effects.

The proposed quality measures may also be useful in other applications of microarray experiments. For example, in microarray meta-analysis we could weight based on not just the quality of a study [24], but the quality of each measurement [25,26].

## Methods

### Quality measures

The quality of a probeset may depend on many quantities such as spatial arrangement on arrays, upper and lower threshold effects, etc. Here we focus on measuring reliability and consistency of a probeset's expression using the probeset's detection p-value. The distribution of expression within a probeset, leading to a detection p-value, is influenced by all stages of the microarray process including scanner brightness, background, RNA quality, chip design, etc. [5]. Therefore, it can be thought as a synthesis index to represent the probeset's quality.

In this paper, we defined quality measures hierarchically at 3 levels (Table 1): (a) gene and array level, (b) gene and group level, summarizing across arrays within each group, (c) gene level, summarizing across arrays as well as groups. Suppose there are *i *= 1, 2, ..., *I *arrays in total, each containing *g *= 1, 2, ..., *G *genes. Further, assume that there are *W *treatment groups, each consisting of *n*_*w *_arrays, for *w *= 1, 2, ..., *W*. Therefore, for the first aspect (a), we measured the quality of the measure of expression for one transcript based on the detection p-value or the Affymetrix Present/Absent/Marginal call [5]. Two measures were defined, which we denote by *q*_*gi*_, the detection p-value, and qgi*
 MathType@MTEF@5@5@+=feaafiart1ev1aaatCvAUfKttLearuWrP9MDH5MBPbIqV92AaeXatLxBI9gBaebbnrfifHhDYfgasaacH8akY=wiFfYdH8Gipec8Eeeu0xXdbba9frFj0=OqFfea0dXdd9vqai=hGuQ8kuc9pgc9s8qqaq=dirpe0xb9q8qiLsFr0=vr0=vr0dc8meaabaqaciaacaGaaeqabaqabeGadaaakeaacqWGXbqCdaqhaaWcbaGaem4zaCMaemyAaKgabaGaeiOkaOcaaaaa@31D2@, based on the present/absent call (see Table 1).

Approaches for summarizing across a set of arrays take two forms. Firstly, the quality of a gene's measurement across a particular group of arrays can be defined; we call this Rgw
 MathType@MTEF@5@5@+=feaafiart1ev1aaatCvAUfKttLearuWrP9MDH5MBPbIqV92AaeXatLxBI9gBaebbnrfifHhDYfgasaacH8akY=wiFfYdH8Gipec8Eeeu0xXdbba9frFj0=OqFfea0dXdd9vqai=hGuQ8kuc9pgc9s8qqaq=dirpe0xb9q8qiLsFr0=vr0=vr0dc8meaabaqaciaacaGaaeqabaqabeGadaaakeaacqWGsbGudaqhaaWcbaGaem4zaCgabaGaem4DaChaaaaa@30D4@. Several different quality scores are described in Table 1, with more detail below for the exponential and beta distributions. Secondly, group-level quality scores can be used to create a single summary measure that applies to all arrays being analyzed. For the latter, we use *Q*_*g *_= max_*w*∈{1 ...*W*}_[Rgw
 MathType@MTEF@5@5@+=feaafiart1ev1aaatCvAUfKttLearuWrP9MDH5MBPbIqV92AaeXatLxBI9gBaebbnrfifHhDYfgasaacH8akY=wiFfYdH8Gipec8Eeeu0xXdbba9frFj0=OqFfea0dXdd9vqai=hGuQ8kuc9pgc9s8qqaq=dirpe0xb9q8qiLsFr0=vr0=vr0dc8meaabaqaciaacaGaaeqabaqabeGadaaakeaacqWGsbGudaqhaaWcbaGaem4zaCgabaGaem4DaChaaaaa@30D4@] . Use of the maximum leads to the desirable property that genes present under one set of experimental conditions but absent under another will be retained for analysis with a high quality score.

To develop a model-based quality measure, we argued as follows: if a gene is not expressed or cannot be measured, then the detection p-values (*q*_*gi*_) are expected to follow a uniform distribution. Equivalently, if a gene's expression cannot be detected, then we can assume a common distribution for this gene for all arrays in group *w*, such that -log(*q*_*gi*_) ~ *Exp*(1). That is, the negative log(*q*_*gi*_) follows an exponential distribution with a rate parameter value of 1 [25]. We therefore made the assumption that the *q*_*gi *_for gene *g *and array *i *follows the one-parameter exponential distribution with a group specific mean *λ*_*gw*_, *w *= 1, ..., *W*, so that -log(*q*_*gi*_) ~ *Exp*(*λ*_*gw*_). The maximum likelihood estimate of *λ*_*gw *_is the inverse of the group-specific sample mean. Define *ν*, a sensitivity parameter, to be a desired threshold for the detection p-values, representing 1 minus the probability that any probeset in a particular treatment group shows a detectable signal. Then *P*(*q*_*giw *_≤ *ν*) leads to Rgw=P(−log⁡(qgiw)≥−log⁡ν)=eλ^gwlog⁡ν
 MathType@MTEF@5@5@+=feaafiart1ev1aaatCvAUfKttLearuWrP9MDH5MBPbIqV92AaeXatLxBI9gBaebbnrfifHhDYfgasaacH8akY=wiFfYdH8Gipec8Eeeu0xXdbba9frFj0=OqFfea0dXdd9vqai=hGuQ8kuc9pgc9s8qqaq=dirpe0xb9q8qiLsFr0=vr0=vr0dc8meaabaqaciaacaGaaeqabaqabeGadaaakeaacqWGsbGudaqhaaWcbaGaem4zaCgabaGaem4DaChaaOGaeyypa0JaemiuaaLaeiikaGIaeyOeI0IagiiBaWMaei4Ba8Maei4zaCMaeiikaGIaemyCae3aaSbaaSqaaiabdEgaNjabdMgaPjabdEha3bqabaGccqGGPaqkcqGHLjYScqGHsislcyGGSbaBcqGGVbWBcqGGNbWziiGacqWF9oGBcqGGPaqkcqGH9aqpcqWGLbqzdaahaaWcbeqaaiqb=T7aSzaajaWaaSbaaWqaaiabdEgaNjabdEha3bqabaWccyGGSbaBcqGGVbWBcqGGNbWzcqWF9oGBaaaaaa@56F2@. Although the distribution of the *q*_*gi *_may not follow an exponential distribution exactly, this simple assumption may give adequate results for developing quality measures.

Theoretically, p-values are expected to follow a two-parameter Beta distribution. However, estimating these two parameters is occasionally impossible due to the fact that the detection p-values are derived from rank tests, and all arrays may have exactly the same p-value when genes are highly expressed. Pounds and Cheng [11] recently proposed a one-parameter Beta distribution to model detection p-values within each experimental group, Rgw=p(qgiw≤ν)=∫0νBeta(q;a^gw)dq=νa^gw
MathType@MTEF@5@5@+=feaafiart1ev1aaatCvAUfKttLearuWrP9MDH5MBPbIqV92AaeXatLxBI9gBaebbnrfifHhDYfgasaacH8akY=wiFfYdH8Gipec8Eeeu0xXdbba9frFj0=OqFfea0dXdd9vqai=hGuQ8kuc9pgc9s8qqaq=dirpe0xb9q8qiLsFr0=vr0=vr0dc8meaabaqaciaacaGaaeqabaqabeGadaaakeaacqWGsbGudaqhaaWcbaGaem4zaCgabaGaem4DaChaaOGaeyypa0JaemiCaaNaeiikaGIaemyCae3aaSbaaSqaaiabdEgaNjabdMgaPjabdEha3bqabaGccqGHKjYOiiGacqWF9oGBcqGGPaqkcqGH9aqpdaWdXbqaaiabdkeacjabdwgaLjabdsha0jabdggaHjabcIcaOiabdghaXjabcUda7iqbdggaHzaajaWaaSbaaSqaaiabdEgaNjabdEha3bqabaGccqGGPaqkcqWGKbazcqWGXbqCaSqaaiabicdaWaqaaiab=17aUbqdcqGHRiI8aOGaeyypa0Jae8xVd42aaWbaaSqabeaacuWGHbqygaqcaiabdEgaNjabdEha3baaaaa@5BB8@, with sensitivity parameter *ν*. The parameter a^gw
 MathType@MTEF@5@5@+=feaafiart1ev1aaatCvAUfKttLearuWrP9MDH5MBPbIqV92AaeXatLxBI9gBaebbnrfifHhDYfgasaacH8akY=wiFfYdH8Gipec8Eeeu0xXdbba9frFj0=OqFfea0dXdd9vqai=hGuQ8kuc9pgc9s8qqaq=dirpe0xb9q8qiLsFr0=vr0=vr0dc8meaabaqaciaacaGaaeqabaqabeGadaaakeaacuWGHbqygaqcamaaBaaaleaacqWGNbWzcqWG3bWDaeqaaaaa@3101@ can be estimated by a^gw=q¯gw/(1−q¯gw)
 MathType@MTEF@5@5@+=feaafiart1ev1aaatCvAUfKttLearuWrP9MDH5MBPbIqV92AaeXatLxBI9gBaebbnrfifHhDYfgasaacH8akY=wiFfYdH8Gipec8Eeeu0xXdbba9frFj0=OqFfea0dXdd9vqai=hGuQ8kuc9pgc9s8qqaq=dirpe0xb9q8qiLsFr0=vr0=vr0dc8meaabaqaciaacaGaaeqabaqabeGadaaakeaacuWGHbqygaqcamaaBaaaleaacqWGNbWzcqWG3bWDaeqaaOGaeyypa0JafmyCaeNbaebadaWgaaWcbaGaem4zaCMaem4DaChabeaakiabc+caViabcIcaOiabigdaXiabgkHiTiqbdghaXzaaraWaaSbaaSqaaiabdEgaNjabdEha3bqabaGccqGGPaqkaaa@3F94@[27], where q¯gw=∑i∈wqgiw/nw
 MathType@MTEF@5@5@+=feaafiart1ev1aaatCvAUfKttLearuWrP9MDH5MBPbIqV92AaeXatLxBI9gBaebbnrfifHhDYfgasaacH8akY=wiFfYdH8Gipec8Eeeu0xXdbba9frFj0=OqFfea0dXdd9vqai=hGuQ8kuc9pgc9s8qqaq=dirpe0xb9q8qiLsFr0=vr0=vr0dc8meaabaqaciaacaGaaeqabaqabeGadaaakeaacuWGXbqCgaqeamaaBaaaleaacqWGNbWzcqWG3bWDaeqaaOGaeyypa0ZaaSGbaeaadaaeqbqaaiabdghaXnaaBaaaleaacqWGNbWzcqWGPbqAcqWG3bWDaeqaaaqaaiabdMgaPjabgIGiolabdEha3bqab0GaeyyeIuoaaOqaaiabd6gaUnaaBaaaleaacqWG3bWDaeqaaaaaaaa@418F@

### Tests of differential expression with quality weights

In traditional meta-analysis, quality measures are often used when combining results from different studies [28]. Without loss of generality, we can assume that we are comparing two groups of microarrays (with *N*_*A *_arrays in group *A *and *N*_*B *_in group *B*), and testing for differentially-expressed genes with the two-sample Welch t-statistic, not assuming equal variances. For gene *g*, the test statistic is therefore tg=x¯gA−x¯gBsgA2/NA+sgB2/NB
 MathType@MTEF@5@5@+=feaafiart1ev1aaatCvAUfKttLearuWrP9MDH5MBPbIqV92AaeXatLxBI9gBaebbnrfifHhDYfgasaacH8akY=wiFfYdH8Gipec8Eeeu0xXdbba9frFj0=OqFfea0dXdd9vqai=hGuQ8kuc9pgc9s8qqaq=dirpe0xb9q8qiLsFr0=vr0=vr0dc8meaabaqaciaacaGaaeqabaqabeGadaaakeaacqWG0baDdaWgaaWcbaGaem4zaCgabeaakiabg2da9maalaaabaGafmiEaGNbaebadaWgaaWcbaGaem4zaCMaemyqaeeabeaakiabgkHiTiqbdIha4zaaraWaaSbaaSqaaiabdEgaNjabdkeacbqabaaakeaadaGcaaqaaiabdohaZnaaDaaaleaacqWGNbWzcqWGbbqqaeaacqaIYaGmaaGccqGGVaWlcqWGobGtdaWgaaWcbaGaemyqaeeabeaakiabgUcaRiabdohaZnaaDaaaleaacqWGNbWzcqWGcbGqaeaacqaIYaGmaaGccqGGVaWlcqWGobGtdaWgaaWcbaGaemOqaieabeaaaeqaaaaaaaa@4B79@, where x¯gA
 MathType@MTEF@5@5@+=feaafiart1ev1aaatCvAUfKttLearuWrP9MDH5MBPbIqV92AaeXatLxBI9gBaebbnrfifHhDYfgasaacH8akY=wiFfYdH8Gipec8Eeeu0xXdbba9frFj0=OqFfea0dXdd9vqai=hGuQ8kuc9pgc9s8qqaq=dirpe0xb9q8qiLsFr0=vr0=vr0dc8meaabaqaciaacaGaaeqabaqabeGadaaakeaacuWG4baEgaqeamaaBaaaleaacqWGNbWzcqWGbbqqaeqaaaaa@30CB@ and x¯gB
 MathType@MTEF@5@5@+=feaafiart1ev1aaatCvAUfKttLearuWrP9MDH5MBPbIqV92AaeXatLxBI9gBaebbnrfifHhDYfgasaacH8akY=wiFfYdH8Gipec8Eeeu0xXdbba9frFj0=OqFfea0dXdd9vqai=hGuQ8kuc9pgc9s8qqaq=dirpe0xb9q8qiLsFr0=vr0=vr0dc8meaabaqaciaacaGaaeqabaqabeGadaaakeaacuWG4baEgaqeamaaBaaaleaacqWGNbWzcqWGcbGqaeqaaaaa@30CD@ denote the sample average intensities in groups *A *and *B*, respectively, and sgA2
 MathType@MTEF@5@5@+=feaafiart1ev1aaatCvAUfKttLearuWrP9MDH5MBPbIqV92AaeXatLxBI9gBaebbnrfifHhDYfgasaacH8akY=wiFfYdH8Gipec8Eeeu0xXdbba9frFj0=OqFfea0dXdd9vqai=hGuQ8kuc9pgc9s8qqaq=dirpe0xb9q8qiLsFr0=vr0=vr0dc8meaabaqaciaacaGaaeqabaqabeGadaaakeaacqWGZbWCdaqhaaWcbaGaem4zaCMaemyqaeeabaGaeGOmaidaaaaa@319C@ and sgB2
 MathType@MTEF@5@5@+=feaafiart1ev1aaatCvAUfKttLearuWrP9MDH5MBPbIqV92AaeXatLxBI9gBaebbnrfifHhDYfgasaacH8akY=wiFfYdH8Gipec8Eeeu0xXdbba9frFj0=OqFfea0dXdd9vqai=hGuQ8kuc9pgc9s8qqaq=dirpe0xb9q8qiLsFr0=vr0=vr0dc8meaabaqaciaacaGaaeqabaqabeGadaaakeaacqWGZbWCdaqhaaWcbaGaem4zaCMaemOqaieabaGaeGOmaidaaaaa@319E@ denote the corresponding sample variances. For more than two groups, an *F *statistic could be used instead. The quality measure (*Q*_*g*_) for gene *g *is then incorporated by tg*
 MathType@MTEF@5@5@+=feaafiart1ev1aaatCvAUfKttLearuWrP9MDH5MBPbIqV92AaeXatLxBI9gBaebbnrfifHhDYfgasaacH8akY=wiFfYdH8Gipec8Eeeu0xXdbba9frFj0=OqFfea0dXdd9vqai=hGuQ8kuc9pgc9s8qqaq=dirpe0xb9q8qiLsFr0=vr0=vr0dc8meaabaqaciaacaGaaeqabaqabeGadaaakeaacqWG0baDdaqhaaWcbaGaem4zaCgabaGaeiOkaOcaaaaa@307D@ = *t*_*g*_*Q*_*g*_. Therefore, tg*
 MathType@MTEF@5@5@+=feaafiart1ev1aaatCvAUfKttLearuWrP9MDH5MBPbIqV92AaeXatLxBI9gBaebbnrfifHhDYfgasaacH8akY=wiFfYdH8Gipec8Eeeu0xXdbba9frFj0=OqFfea0dXdd9vqai=hGuQ8kuc9pgc9s8qqaq=dirpe0xb9q8qiLsFr0=vr0=vr0dc8meaabaqaciaacaGaaeqabaqabeGadaaakeaacqWG0baDdaqhaaWcbaGaem4zaCgabaGaeiOkaOcaaaaa@307D@ = *t*_*g *_when *Q*_*g *_= 1, and tg*
 MathType@MTEF@5@5@+=feaafiart1ev1aaatCvAUfKttLearuWrP9MDH5MBPbIqV92AaeXatLxBI9gBaebbnrfifHhDYfgasaacH8akY=wiFfYdH8Gipec8Eeeu0xXdbba9frFj0=OqFfea0dXdd9vqai=hGuQ8kuc9pgc9s8qqaq=dirpe0xb9q8qiLsFr0=vr0=vr0dc8meaabaqaciaacaGaaeqabaqabeGadaaakeaacqWG0baDdaqhaaWcbaGaem4zaCgabaGaeiOkaOcaaaaa@307D@ goes to zero with low quality scores.

We converted this modified t-test statistic to a p-value by reference to a standard t-distribution with degrees of freedom based on Satterthwaite's approximation [29], assuming unequal variances between groups *A *and *B*. Of course, it is clear that tg*
 MathType@MTEF@5@5@+=feaafiart1ev1aaatCvAUfKttLearuWrP9MDH5MBPbIqV92AaeXatLxBI9gBaebbnrfifHhDYfgasaacH8akY=wiFfYdH8Gipec8Eeeu0xXdbba9frFj0=OqFfea0dXdd9vqai=hGuQ8kuc9pgc9s8qqaq=dirpe0xb9q8qiLsFr0=vr0=vr0dc8meaabaqaciaacaGaaeqabaqabeGadaaakeaacqWG0baDdaqhaaWcbaGaem4zaCgabaGaeiOkaOcaaaaa@307D@ will no longer follow the t-distribution, since the kurtosis will be substantially altered as *Q*_*g *_gets closer to zero. However, the next section describes how these altered p-values were used in modified adjustments for multiple testing.

This simple approach of weighting the t-test statistics can be contrasted with the approach of weighting the expression intensities within the test statistic. Using standard formula for weighting based on quality, we constructed tqg=x¯qgA−x¯qgBsqgA2/NA+sqgB2/NB
 MathType@MTEF@5@5@+=feaafiart1ev1aaatCvAUfKttLearuWrP9MDH5MBPbIqV92AaeXatLxBI9gBaebbnrfifHhDYfgasaacH8akY=wiFfYdH8Gipec8Eeeu0xXdbba9frFj0=OqFfea0dXdd9vqai=hGuQ8kuc9pgc9s8qqaq=dirpe0xb9q8qiLsFr0=vr0=vr0dc8meaabaqaciaacaGaaeqabaqabeGadaaakeaacqWG0baDdaWgaaWcbaGaemyCaeNaem4zaCgabeaakiabg2da9maalaaabaGafmiEaGNbaebadaWgaaWcbaGaemyCaeNaem4zaCMaemyqaeeabeaakiabgkHiTiqbdIha4zaaraWaaSbaaSqaaiabdghaXjabdEgaNjabdkeacbqabaaakeaadaGcaaqaaiabdohaZnaaDaaaleaacqWGXbqCcqWGNbWzcqWGbbqqaeaacqaIYaGmaaGccqGGVaWlcqWGobGtdaWgaaWcbaGaemyqaeeabeaakiabgUcaRiabdohaZnaaDaaaleaacqWGXbqCcqWGNbWzcqWGcbGqaeaacqaIYaGmaaGccqGGVaWlcqWGobGtdaWgaaWcbaGaemOqaieabeaaaeqaaaaaaaa@5290@, where x¯qgw
 MathType@MTEF@5@5@+=feaafiart1ev1aaatCvAUfKttLearuWrP9MDH5MBPbIqV92AaeXatLxBI9gBaebbnrfifHhDYfgasaacH8akY=wiFfYdH8Gipec8Eeeu0xXdbba9frFj0=OqFfea0dXdd9vqai=hGuQ8kuc9pgc9s8qqaq=dirpe0xb9q8qiLsFr0=vr0=vr0dc8meaabaqaciaacaGaaeqabaqabeGadaaakeaacuWG4baEgaqeamaaBaaaleaacqWGXbqCcqWGNbWzcqWG3bWDaeqaaaaa@32A2@ and sqgw2
 MathType@MTEF@5@5@+=feaafiart1ev1aaatCvAUfKttLearuWrP9MDH5MBPbIqV92AaeXatLxBI9gBaebbnrfifHhDYfgasaacH8akY=wiFfYdH8Gipec8Eeeu0xXdbba9frFj0=OqFfea0dXdd9vqai=hGuQ8kuc9pgc9s8qqaq=dirpe0xb9q8qiLsFr0=vr0=vr0dc8meaabaqaciaacaGaaeqabaqabeGadaaakeaacqWGZbWCdaqhaaWcbaGaemyCaeNaem4zaCMaem4DaChabaGaeGOmaidaaaaa@3373@ are the group-specific weighted means and standard deviations [30], weighted by 1 - *q*_*qi*_. Here, *w *= *A*, *B*. We obtained *p*_*qtg *_from a *t*-distribution with degrees of freedom based on Satterthwaite's approximation [29]. For brevity, we refer to this approach as Qgwe
 MathType@MTEF@5@5@+=feaafiart1ev1aaatCvAUfKttLearuWrP9MDH5MBPbIqV92AaeXatLxBI9gBaebbnrfifHhDYfgasaacH8akY=wiFfYdH8Gipec8Eeeu0xXdbba9frFj0=OqFfea0dXdd9vqai=hGuQ8kuc9pgc9s8qqaq=dirpe0xb9q8qiLsFr0=vr0=vr0dc8meaabaqaciaacaGaaeqabaqabeGadaaakeaacqWGrbqudaqhaaWcbaGaem4zaCgabaGaem4DaCNaemyzaugaaaaa@3225@ even though there was no specific quality measure attached to each gene.

### Multiple hypotheses testing with weights

Several authors have considered the problem of including weights in multiple hypothesis testing situations [31-33]. Often, weights have been introduced when some of the hypotheses *H*_*i *_are deemed more important than others. For example, Holm [31] first introduced weights into his sequentially rejective multiple hypothesis testing procedure, where weight was used to indicate the importance of the hypotheses. In that spirit, testing for differences in genes that are not expressed or non-specific in such genes should be considered less important than testing for differences among genes that are specific and well-expressed.

To give another perspective, our quality-weighted statistics t* can also be considered as an implementation of a filtering method. For example, Qgcall
 MathType@MTEF@5@5@+=feaafiart1ev1aaatCvAUfKttLearuWrP9MDH5MBPbIqV92AaeXatLxBI9gBaebbnrfifHhDYfgasaacH8akY=wiFfYdH8Gipec8Eeeu0xXdbba9frFj0=OqFfea0dXdd9vqai=hGuQ8kuc9pgc9s8qqaq=dirpe0xb9q8qiLsFr0=vr0=vr0dc8meaabaqaciaacaGaaeqabaqabeGadaaakeaacqWGrbqudaqhaaWcbaGaem4zaCgabaGaem4yamMaemyyaeMaemiBaWMaemiBaWgaaaaa@34B7@ is a straightforward implementation of filtering based on including only genes with present calls on all arrays, and our other measures effectively exclude genes with small quality measures when the weighted statistic t* is close to zero. Therefore, we propose to adjust the effective number of genes tested to correspond to the number of tested genes of high quality.

Assume that quality measures *Q*_1_, *Q*_2_, ..., *Q*_*G *_and significance values *p*_1_, *p*_2_, ..., *p*_*G *_have been calculated for all G genes. Let p1*≤p2*≤,…,≤pG*
 MathType@MTEF@5@5@+=feaafiart1ev1aaatCvAUfKttLearuWrP9MDH5MBPbIqV92AaeXatLxBI9gBaebbnrfifHhDYfgasaacH8akY=wiFfYdH8Gipec8Eeeu0xXdbba9frFj0=OqFfea0dXdd9vqai=hGuQ8kuc9pgc9s8qqaq=dirpe0xb9q8qiLsFr0=vr0=vr0dc8meaabaqaciaacaGaaeqabaqabeGadaaakeaacqWGWbaCdaqhaaWcbaGaeGymaedabaGaeiOkaOcaaOGaeyizImQaemiCaa3aa0baaSqaaiabikdaYaqaaiabcQcaQaaakiabgsMiJkabcYcaSiablAciljabcYcaSiabgsMiJkabdchaWnaaDaaaleaacqWGhbWraeaacqGGQaGkaaaaaa@3F10@ denote the ordered significance values, and let Q1*,Q2*,…,QG*
 MathType@MTEF@5@5@+=feaafiart1ev1aaatCvAUfKttLearuWrP9MDH5MBPbIqV92AaeXatLxBI9gBaebbnrfifHhDYfgasaacH8akY=wiFfYdH8Gipec8Eeeu0xXdbba9frFj0=OqFfea0dXdd9vqai=hGuQ8kuc9pgc9s8qqaq=dirpe0xb9q8qiLsFr0=vr0=vr0dc8meaabaqaciaacaGaaeqabaqabeGadaaakeaacqWGrbqudaqhaaWcbaGaeGymaedabaGaeiOkaOcaaOGaeiilaWIaemyuae1aa0baaSqaaiabikdaYaqaaiabcQcaQaaakiabcYcaSiablAciljabcYcaSiabdgfarnaaDaaaleaacqWGhbWraeaacqGGQaGkaaaaaa@3A17@ denote the quality measures in the same order. We redefined the Benjamini and Hochberg (BH) multiple testing procedure as p˜gWBH=min⁡u=g,…,G{(min⁡(∑z=1GQz*∑z=1uQz*pu*,1)}
 MathType@MTEF@5@5@+=feaafiart1ev1aaatCvAUfKttLearuWrP9MDH5MBPbIqV92AaeXatLxBI9gBaebbnrfifHhDYfgasaacH8akY=wiFfYdH8Gipec8Eeeu0xXdbba9frFj0=OqFfea0dXdd9vqai=hGuQ8kuc9pgc9s8qqaq=dirpe0xb9q8qiLsFr0=vr0=vr0dc8meaabaqaciaacaGaaeqabaqabeGadaaakeaacuWGWbaCgaacamaaDaaaleaacqWGNbWzaeaacqWGxbWvcqWGcbGqcqWGibasaaGccqGH9aqpcyGGTbqBcqGGPbqAcqGGUbGBdaWgaaWcbaGaemyDauNaeyypa0Jaem4zaCMaeiilaWIaeSOjGSKaeiilaWIaem4raCeabeaakiabcUha7jabcIcaOiGbc2gaTjabcMgaPjabc6gaUjabcIcaOmaalaaabaWaaabCaeaacqWGrbqudaqhaaWcbaGaemOEaOhabaGaeiOkaOcaaaqaaiabdQha6jabg2da9iabigdaXaqaaiabdEeahbqdcqGHris5aaGcbaWaaabCaeaacqWGrbqudaqhaaWcbaGaemOEaOhabaGaeiOkaOcaaaqaaiabdQha6jabg2da9iabigdaXaqaaiabdwha1bqdcqGHris5aaaakiabdchaWnaaDaaaleaacqWG1bqDaeaacqGGQaGkaaGccqGGSaalcqaIXaqmcqGGPaqkcqGG9bqFaaa@6505@. The sums of the quality measures in this formula estimate the number of high quality genes instead of the number of genes. We call this approach the Weighted Benjamini and Hochberg (WBH) method. Similar modifications lead to a weighted Benjamini and Yekutieli (WBY) procedure (not shown).

We applied WBH to the t-statistics modified by the quality measures Qgcall
 MathType@MTEF@5@5@+=feaafiart1ev1aaatCvAUfKttLearuWrP9MDH5MBPbIqV92AaeXatLxBI9gBaebbnrfifHhDYfgasaacH8akY=wiFfYdH8Gipec8Eeeu0xXdbba9frFj0=OqFfea0dXdd9vqai=hGuQ8kuc9pgc9s8qqaq=dirpe0xb9q8qiLsFr0=vr0=vr0dc8meaabaqaciaacaGaaeqabaqabeGadaaakeaacqWGrbqudaqhaaWcbaGaem4zaCgabaGaem4yamMaemyyaeMaemiBaWMaemiBaWgaaaaa@34B7@, Qgexp⁡
 MathType@MTEF@5@5@+=feaafiart1ev1aaatCvAUfKttLearuWrP9MDH5MBPbIqV92AaeXatLxBI9gBaebbnrfifHhDYfgasaacH8akY=wiFfYdH8Gipec8Eeeu0xXdbba9frFj0=OqFfea0dXdd9vqai=hGuQ8kuc9pgc9s8qqaq=dirpe0xb9q8qiLsFr0=vr0=vr0dc8meaabaqaciaacaGaaeqabaqabeGadaaakeaacqWGrbqudaqhaaWcbaGaem4zaCgabaGagiyzauMaeiiEaGNaeiiCaahaaaaa@338F@, Qgbeta
 MathType@MTEF@5@5@+=feaafiart1ev1aaatCvAUfKttLearuWrP9MDH5MBPbIqV92AaeXatLxBI9gBaebbnrfifHhDYfgasaacH8akY=wiFfYdH8Gipec8Eeeu0xXdbba9frFj0=OqFfea0dXdd9vqai=hGuQ8kuc9pgc9s8qqaq=dirpe0xb9q8qiLsFr0=vr0=vr0dc8meaabaqaciaacaGaaeqabaqabeGadaaakeaacqWGrbqudaqhaaWcbaGaem4zaCgabaGaemOyaiMaemyzauMaemiDaqNaemyyaegaaaaa@34B7@ and Qgmeanp
 MathType@MTEF@5@5@+=feaafiart1ev1aaatCvAUfKttLearuWrP9MDH5MBPbIqV92AaeXatLxBI9gBaebbnrfifHhDYfgasaacH8akY=wiFfYdH8Gipec8Eeeu0xXdbba9frFj0=OqFfea0dXdd9vqai=hGuQ8kuc9pgc9s8qqaq=dirpe0xb9q8qiLsFr0=vr0=vr0dc8meaabaqaciaacaGaaeqabaqabeGadaaakeaacqWGrbqudaqhaaWcbaGaem4zaCgabaGaemyBa0MaemyzauMaemyyaeMaemOBa4MaemiCaahaaaaa@362A@. For the t-statistics modified by quality measures Qg0
 MathType@MTEF@5@5@+=feaafiart1ev1aaatCvAUfKttLearuWrP9MDH5MBPbIqV92AaeXatLxBI9gBaebbnrfifHhDYfgasaacH8akY=wiFfYdH8Gipec8Eeeu0xXdbba9frFj0=OqFfea0dXdd9vqai=hGuQ8kuc9pgc9s8qqaq=dirpe0xb9q8qiLsFr0=vr0=vr0dc8meaabaqaciaacaGaaeqabaqabeGadaaakeaacqWGrbqudaqhaaWcbaGaem4zaCgabaGaeGimaadaaaaa@3049@ and Qgwe
 MathType@MTEF@5@5@+=feaafiart1ev1aaatCvAUfKttLearuWrP9MDH5MBPbIqV92AaeXatLxBI9gBaebbnrfifHhDYfgasaacH8akY=wiFfYdH8Gipec8Eeeu0xXdbba9frFj0=OqFfea0dXdd9vqai=hGuQ8kuc9pgc9s8qqaq=dirpe0xb9q8qiLsFr0=vr0=vr0dc8meaabaqaciaacaGaaeqabaqabeGadaaakeaacqWGrbqudaqhaaWcbaGaem4zaCgabaGaem4DaCNaemyzaugaaaaa@3225@ as well as local pooled error (LPE) test QgLPE
 MathType@MTEF@5@5@+=feaafiart1ev1aaatCvAUfKttLearuWrP9MDH5MBPbIqV92AaeXatLxBI9gBaebbnrfifHhDYfgasaacH8akY=wiFfYdH8Gipec8Eeeu0xXdbba9frFj0=OqFfea0dXdd9vqai=hGuQ8kuc9pgc9s8qqaq=dirpe0xb9q8qiLsFr0=vr0=vr0dc8meaabaqaciaacaGaaeqabaqabeGadaaakeaacqWGrbqudaqhaaWcbaGaem4zaCgabaGaemitaWKaemiuaaLaemyraueaaaaa@32B8@, the standard form of the Benjamini and Hochberg (BH) multiple testing procedure was used.

### Simulation method

Affymetrix probe level data were generated based on a unified model proposed by Wu et al. [14]. When treatment effects are considered for different conditions, such as cancer and normal tissues, gene expression across these conditions can be modeled as:

Ygij=OgijPM+NgijPM+SgijPM=OgijPM+exp⁡(μgijPM+εgijPM)+exp⁡(γg+δgXi+agij+bi+ζgijPM)Wgij=OgijMM+NgijMM+ΦSgijMM=OgijMM+exp⁡(μgijMM+εgijMM)+Φgjexp⁡(γg+δgXi+agij+bi+ζgijMM),     (2)
 MathType@MTEF@5@5@+=feaafiart1ev1aaatCvAUfKttLearuWrP9MDH5MBPbIqV92AaeXatLxBI9gBaebbnrfifHhDYfgasaacH8akY=wiFfYdH8Gipec8Eeeu0xXdbba9frFj0=OqFfea0dXdd9vqai=hGuQ8kuc9pgc9s8qqaq=dirpe0xb9q8qiLsFr0=vr0=vr0dc8meaabaqaciaacaGaaeqabaqabeGadaaakeaafaqadeabbaaaaeaacqWGzbqwdaWgaaWcbaGaem4zaCMaemyAaKMaemOAaOgabeaakiabg2da9iabd+eapnaaDaaaleaacqWGNbWzcqWGPbqAcqWGQbGAaeaacqWGqbaucqWGnbqtaaGccqGHRaWkcqWGobGtdaqhaaWcbaGaem4zaCMaemyAaKMaemOAaOgabaGaemiuaaLaemyta0eaaOGaey4kaSIaem4uam1aa0baaSqaaiabdEgaNjabdMgaPjabdQgaQbqaaiabdcfaqjabd2eanbaaaOqaaiabg2da9iabd+eapnaaDaaaleaacqWGNbWzcqWGPbqAcqWGQbGAaeaacqWGqbaucqWGnbqtaaGccqGHRaWkcyGGLbqzcqGG4baEcqGGWbaCcqGGOaakiiGacqWF8oqBdaqhaaWcbaGaem4zaCMaemyAaKMaemOAaOgabaGaemiuaaLaemyta0eaaOGaey4kaSIae8xTdu2aa0baaSqaaiabdEgaNjabdMgaPjabdQgaQbqaaiabdcfaqjabd2eanbaakiabcMcaPiabgUcaRiGbcwgaLjabcIha4jabcchaWjabcIcaOiab=n7aNnaaBaaaleaacqWGNbWzaeqaaOGaey4kaSIae8hTdq2aaSbaaSqaaiabdEgaNbqabaGccqWGybawdaWgaaWcbaGaemyAaKgabeaakiabgUcaRiabdggaHnaaBaaaleaacqWGNbWzcqWGPbqAcqWGQbGAaeqaaOGaey4kaSIaemOyai2aaSbaaSqaaiabdMgaPbqabaGccqGHRaWkcqWF2oGEdaqhaaWcbaGaem4zaCMaemyAaKMaemOAaOgabaGaemiuaaLaemyta0eaaOGaeiykaKcabaGaem4vaC1aaSbaaSqaaiabdEgaNjabdMgaPjabdQgaQbqabaGccqGH9aqpcqWGpbWtdaqhaaWcbaGaem4zaCMaemyAaKMaemOAaOgabaGaemyta0Kaemyta0eaaOGaey4kaSIaemOta40aa0baaSqaaiabdEgaNjabdMgaPjabdQgaQbqaaiabd2eanjabd2eanbaakiabgUcaRiabfA6agjabdofatnaaDaaaleaacqWGNbWzcqWGPbqAcqWGQbGAaeaacqWGnbqtcqWGnbqtaaaakeaacqGH9aqpcqWGpbWtdaqhaaWcbaGaem4zaCMaemyAaKMaemOAaOgabaGaemyta0Kaemyta0eaaOGaey4kaSIagiyzauMaeiiEaGNaeiiCaaNaeiikaGIae8hVd02aa0baaSqaaiabdEgaNjabdMgaPjabdQgaQbqaaiabd2eanjabd2eanbaakiabgUcaRiab=v7aLnaaDaaaleaacqWGNbWzcqWGPbqAcqWGQbGAaeaacqWGnbqtcqWGnbqtaaGccqGGPaqkcqGHRaWkcqqHMoGrdaWgaaWcbaGaem4zaCMaemOAaOgabeaakiGbcwgaLjabcIha4jabcchaWjabcIcaOiab=n7aNnaaBaaaleaacqWGNbWzaeqaaOGaey4kaSIae8hTdq2aaSbaaSqaaiabdEgaNbqabaGccqWGybawdaWgaaWcbaGaemyAaKgabeaakiabgUcaRiabdggaHnaaBaaaleaacqWGNbWzcqWGPbqAcqWGQbGAaeqaaOGaey4kaSIaemOyai2aaSbaaSqaaiabdMgaPbqabaGccqGHRaWkcqWF2oGEdaqhaaWcbaGaem4zaCMaemyAaKMaemOAaOgabaGaemyta0Kaemyta0eaaOGaeiykaKIaeiilaWcaaiaaxMaacaWLjaWaaeWaaeaacqaIYaGmaiaawIcacaGLPaaaaaa@002C@

where *Y*_*gij *_and *W*_*gij *_are the *PM *and *MM *intensities for the probe *j *in probeset *g *on array *i *respectively; *O *denotes optical noise. *N *represents non-specific binding (NSB) noise. *S *is a quantity proportional to RNA expression and the coefficient 0 < Φ < 1 accounts for the fact that for some probe-pairs the *MM *detects signal. Following Wu et al. [14], we simulated OgijPM
 MathType@MTEF@5@5@+=feaafiart1ev1aaatCvAUfKttLearuWrP9MDH5MBPbIqV92AaeXatLxBI9gBaebbnrfifHhDYfgasaacH8akY=wiFfYdH8Gipec8Eeeu0xXdbba9frFj0=OqFfea0dXdd9vqai=hGuQ8kuc9pgc9s8qqaq=dirpe0xb9q8qiLsFr0=vr0=vr0dc8meaabaqaciaacaGaaeqabaqabeGadaaakeaacqWGpbWtdaqhaaWcbaGaem4zaCMaemyAaKMaemOAaOgabaGaemiuaaLaemyta0eaaaaa@345B@ and OgijMM
 MathType@MTEF@5@5@+=feaafiart1ev1aaatCvAUfKttLearuWrP9MDH5MBPbIqV92AaeXatLxBI9gBaebbnrfifHhDYfgasaacH8akY=wiFfYdH8Gipec8Eeeu0xXdbba9frFj0=OqFfea0dXdd9vqai=hGuQ8kuc9pgc9s8qqaq=dirpe0xb9q8qiLsFr0=vr0=vr0dc8meaabaqaciaacaGaaeqabaqabeGadaaakeaacqWGpbWtdaqhaaWcbaGaem4zaCMaemyAaKMaemOAaOgabaGaemyta0Kaemyta0eaaaaa@3455@ using independent draws from log_2_(OgijPM
 MathType@MTEF@5@5@+=feaafiart1ev1aaatCvAUfKttLearuWrP9MDH5MBPbIqV92AaeXatLxBI9gBaebbnrfifHhDYfgasaacH8akY=wiFfYdH8Gipec8Eeeu0xXdbba9frFj0=OqFfea0dXdd9vqai=hGuQ8kuc9pgc9s8qqaq=dirpe0xb9q8qiLsFr0=vr0=vr0dc8meaabaqaciaacaGaaeqabaqabeGadaaakeaacqWGpbWtdaqhaaWcbaGaem4zaCMaemyAaKMaemOAaOgabaGaemiuaaLaemyta0eaaaaa@345B@) ~ *N*(5, 0.1) and log_2_(OgijMM
 MathType@MTEF@5@5@+=feaafiart1ev1aaatCvAUfKttLearuWrP9MDH5MBPbIqV92AaeXatLxBI9gBaebbnrfifHhDYfgasaacH8akY=wiFfYdH8Gipec8Eeeu0xXdbba9frFj0=OqFfea0dXdd9vqai=hGuQ8kuc9pgc9s8qqaq=dirpe0xb9q8qiLsFr0=vr0=vr0dc8meaabaqaciaacaGaaeqabaqabeGadaaakeaacqWGpbWtdaqhaaWcbaGaem4zaCMaemyAaKMaemOAaOgabaGaemyta0Kaemyta0eaaaaa@3455@) ~ *N*(5, 0.1). We set μgjPM
 MathType@MTEF@5@5@+=feaafiart1ev1aaatCvAUfKttLearuWrP9MDH5MBPbIqV92AaeXatLxBI9gBaebbnrfifHhDYfgasaacH8akY=wiFfYdH8Gipec8Eeeu0xXdbba9frFj0=OqFfea0dXdd9vqai=hGuQ8kuc9pgc9s8qqaq=dirpe0xb9q8qiLsFr0=vr0=vr0dc8meaabaqaciaacaGaaeqabaqabeGadaaakeaaiiGacqWF8oqBdaqhaaWcbaGaem4zaCMaemOAaOgabaGaemiuaaLaemyta0eaaaaa@3396@ = μgjPM
 MathType@MTEF@5@5@+=feaafiart1ev1aaatCvAUfKttLearuWrP9MDH5MBPbIqV92AaeXatLxBI9gBaebbnrfifHhDYfgasaacH8akY=wiFfYdH8Gipec8Eeeu0xXdbba9frFj0=OqFfea0dXdd9vqai=hGuQ8kuc9pgc9s8qqaq=dirpe0xb9q8qiLsFr0=vr0=vr0dc8meaabaqaciaacaGaaeqabaqabeGadaaakeaaiiGacqWF8oqBdaqhaaWcbaGaem4zaCMaemOAaOgabaGaemiuaaLaemyta0eaaaaa@3396@ = 4.6, and assumed that εgjPM
 MathType@MTEF@5@5@+=feaafiart1ev1aaatCvAUfKttLearuWrP9MDH5MBPbIqV92AaeXatLxBI9gBaebbnrfifHhDYfgasaacH8akY=wiFfYdH8Gipec8Eeeu0xXdbba9frFj0=OqFfea0dXdd9vqai=hGuQ8kuc9pgc9s8qqaq=dirpe0xb9q8qiLsFr0=vr0=vr0dc8meaabaqaciaacaGaaeqabaqabeGadaaakeaaiiGacqWF1oqzdaqhaaWcbaGaem4zaCMaemOAaOgabaGaemiuaaLaemyta0eaaaaa@3387@ and εgjMM
 MathType@MTEF@5@5@+=feaafiart1ev1aaatCvAUfKttLearuWrP9MDH5MBPbIqV92AaeXatLxBI9gBaebbnrfifHhDYfgasaacH8akY=wiFfYdH8Gipec8Eeeu0xXdbba9frFj0=OqFfea0dXdd9vqai=hGuQ8kuc9pgc9s8qqaq=dirpe0xb9q8qiLsFr0=vr0=vr0dc8meaabaqaciaacaGaaeqabaqabeGadaaakeaaiiGacqWF1oqzdaqhaaWcbaGaem4zaCMaemOAaOgabaGaemyta0Kaemyta0eaaaaa@3381@ follow a bivariate normal distribution with mean 0, variance 1, and correlation 0.88. We then generated identically and independently distributed random variates *e *~ *N*(0,0.08), so that εgijPM=εgjPM+egijPM
 MathType@MTEF@5@5@+=feaafiart1ev1aaatCvAUfKttLearuWrP9MDH5MBPbIqV92AaeXatLxBI9gBaebbnrfifHhDYfgasaacH8akY=wiFfYdH8Gipec8Eeeu0xXdbba9frFj0=OqFfea0dXdd9vqai=hGuQ8kuc9pgc9s8qqaq=dirpe0xb9q8qiLsFr0=vr0=vr0dc8meaabaqaciaacaGaaeqabaqabeGadaaakeaaiiGacqWF1oqzdaqhaaWcbaGaem4zaCMaemyAaKMaemOAaOgabaGaemiuaaLaemyta0eaaOGaeyypa0Jae8xTdu2aa0baaSqaaiabdEgaNjabdQgaQbqaaiabdcfaqjabd2eanbaakiabgUcaRiabdwgaLnaaDaaaleaacqWGNbWzcqWGPbqAcqWGQbGAaeaacqWGqbaucqWGnbqtaaaaaa@4588@ and similarly εgijMM=εgjMM+egijMM
 MathType@MTEF@5@5@+=feaafiart1ev1aaatCvAUfKttLearuWrP9MDH5MBPbIqV92AaeXatLxBI9gBaebbnrfifHhDYfgasaacH8akY=wiFfYdH8Gipec8Eeeu0xXdbba9frFj0=OqFfea0dXdd9vqai=hGuQ8kuc9pgc9s8qqaq=dirpe0xb9q8qiLsFr0=vr0=vr0dc8meaabaqaciaacaGaaeqabaqabeGadaaakeaaiiGacqWF1oqzdaqhaaWcbaGaem4zaCMaemyAaKMaemOAaOgabaGaemyta0Kaemyta0eaaOGaeyypa0Jae8xTdu2aa0baaSqaaiabdEgaNjabdQgaQbqaaiabd2eanjabd2eanbaakiabgUcaRiabdwgaLnaaDaaaleaacqWGNbWzcqWGPbqAcqWGQbGAaeaacqWGnbqtcqWGnbqtaaaaaa@4576@. When mismatch probe *j *of gene *g *is attached by picking up stray signal, Φ_*gi *_is generated as Φ_*gi *_~ *Beta*(0.5,5), otherwise, Φ_*gi *_= 0. The proportion of attached probes among total probes was set to 0.01. Since *S *follows a power law, we set its base to 2. Therefore, if we denote *γ*_*g *_as the baseline log expression level for probeset *g*, we can select log_2_(*γ*_*g*_) expression levels from 0 to 12, which were generated from *γ*_*g *_~ 12* *Beta*(1,3) + 1. *δ*_*g *_is the expected differential expression of gene *g *across different conditions, which is varied in the simulations. *b*_*i*_, which describes the need for normalization, was set to be zero. *α*_*gij *_is the signal detecting ability of probe *j *in gene *g *on array *i*, which is assumed to follow a normal distribution with mean zero and signal detection variance σα2
 MathType@MTEF@5@5@+=feaafiart1ev1aaatCvAUfKttLearuWrP9MDH5MBPbIqV92AaeXatLxBI9gBaebbnrfifHhDYfgasaacH8akY=wiFfYdH8Gipec8Eeeu0xXdbba9frFj0=OqFfea0dXdd9vqai=hGuQ8kuc9pgc9s8qqaq=dirpe0xb9q8qiLsFr0=vr0=vr0dc8meaabaqaciaacaGaaeqabaqabeGadaaakeaaiiGacqWFdpWCdaqhaaWcbaGae8xSdegabaGaeGOmaidaaaaa@312F@. Multiplicative errors ζgijPM
 MathType@MTEF@5@5@+=feaafiart1ev1aaatCvAUfKttLearuWrP9MDH5MBPbIqV92AaeXatLxBI9gBaebbnrfifHhDYfgasaacH8akY=wiFfYdH8Gipec8Eeeu0xXdbba9frFj0=OqFfea0dXdd9vqai=hGuQ8kuc9pgc9s8qqaq=dirpe0xb9q8qiLsFr0=vr0=vr0dc8meaabaqaciaacaGaaeqabaqabeGadaaakeaaiiGacqWF2oGEdaqhaaWcbaGaem4zaCMaemyAaKMaemOAaOgabaGaemiuaaLaemyta0eaaaaa@34F8@ and ζgijMM
 MathType@MTEF@5@5@+=feaafiart1ev1aaatCvAUfKttLearuWrP9MDH5MBPbIqV92AaeXatLxBI9gBaebbnrfifHhDYfgasaacH8akY=wiFfYdH8Gipec8Eeeu0xXdbba9frFj0=OqFfea0dXdd9vqai=hGuQ8kuc9pgc9s8qqaq=dirpe0xb9q8qiLsFr0=vr0=vr0dc8meaabaqaciaacaGaaeqabaqabeGadaaakeaaiiGacqWF2oGEdaqhaaWcbaGaem4zaCMaemyAaKMaemOAaOgabaGaemyta0Kaemyta0eaaaaa@34F2@ were generated independently from *N*(0, σζ2
 MathType@MTEF@5@5@+=feaafiart1ev1aaatCvAUfKttLearuWrP9MDH5MBPbIqV92AaeXatLxBI9gBaebbnrfifHhDYfgasaacH8akY=wiFfYdH8Gipec8Eeeu0xXdbba9frFj0=OqFfea0dXdd9vqai=hGuQ8kuc9pgc9s8qqaq=dirpe0xb9q8qiLsFr0=vr0=vr0dc8meaabaqaciaacaGaaeqabaqabeGadaaakeaaiiGacqWFdpWCdaqhaaWcbaGae8NTdOhabaGaeGOmaidaaaaa@314D@). Values of σα2
 MathType@MTEF@5@5@+=feaafiart1ev1aaatCvAUfKttLearuWrP9MDH5MBPbIqV92AaeXatLxBI9gBaebbnrfifHhDYfgasaacH8akY=wiFfYdH8Gipec8Eeeu0xXdbba9frFj0=OqFfea0dXdd9vqai=hGuQ8kuc9pgc9s8qqaq=dirpe0xb9q8qiLsFr0=vr0=vr0dc8meaabaqaciaacaGaaeqabaqabeGadaaakeaaiiGacqWFdpWCdaqhaaWcbaGae8xSdegabaGaeGOmaidaaaaa@312F@ and σζ2
 MathType@MTEF@5@5@+=feaafiart1ev1aaatCvAUfKttLearuWrP9MDH5MBPbIqV92AaeXatLxBI9gBaebbnrfifHhDYfgasaacH8akY=wiFfYdH8Gipec8Eeeu0xXdbba9frFj0=OqFfea0dXdd9vqai=hGuQ8kuc9pgc9s8qqaq=dirpe0xb9q8qiLsFr0=vr0=vr0dc8meaabaqaciaacaGaaeqabaqabeGadaaakeaaiiGacqWFdpWCdaqhaaWcbaGae8NTdOhabaGaeGOmaidaaaaa@314D@ were varied in the simulations. Since the theoretical maximum value of an Affymetrix scanner is 2^16^, we kept only generated *Y*_*gij *_and *W*_*gij *_less than 2^16^, that is, Ygij=min⁡(OgijPM+NgijPM+SgijPM,216)
 MathType@MTEF@5@5@+=feaafiart1ev1aaatCvAUfKttLearuWrP9MDH5MBPbIqV92AaeXatLxBI9gBaebbnrfifHhDYfgasaacH8akY=wiFfYdH8Gipec8Eeeu0xXdbba9frFj0=OqFfea0dXdd9vqai=hGuQ8kuc9pgc9s8qqaq=dirpe0xb9q8qiLsFr0=vr0=vr0dc8meaabaqaciaacaGaaeqabaqabeGadaaakeaacqWGzbqwdaWgaaWcbaGaem4zaCMaemyAaKMaemOAaOgabeaakiabg2da9iGbc2gaTjabcMgaPjabc6gaUjabcIcaOiabd+eapnaaDaaaleaacqWGNbWzcqWGPbqAcqWGQbGAaeaacqWGqbaucqWGnbqtaaGccqGHRaWkcqWGobGtdaqhaaWcbaGaem4zaCMaemyAaKMaemOAaOgabaGaemiuaaLaemyta0eaaOGaey4kaSIaem4uam1aa0baaSqaaiabdEgaNjabdMgaPjabdQgaQbqaaiabdcfaqjabd2eanbaakiabcYcaSiabikdaYmaaCaaaleqabaGaeGymaeJaeGOnaydaaOGaeiykaKcaaa@55EE@ and Wgij=min⁡(OgijMM+NgijMM+ΦSgijMM,216)
 MathType@MTEF@5@5@+=feaafiart1ev1aaatCvAUfKttLearuWrP9MDH5MBPbIqV92AaeXatLxBI9gBaebbnrfifHhDYfgasaacH8akY=wiFfYdH8Gipec8Eeeu0xXdbba9frFj0=OqFfea0dXdd9vqai=hGuQ8kuc9pgc9s8qqaq=dirpe0xb9q8qiLsFr0=vr0=vr0dc8meaabaqaciaacaGaaeqabaqabeGadaaakeaacqWGxbWvdaWgaaWcbaGaem4zaCMaemyAaKMaemOAaOgabeaakiabg2da9iGbc2gaTjabcMgaPjabc6gaUjabcIcaOiabd+eapnaaDaaaleaacqWGNbWzcqWGPbqAcqWGQbGAaeaacqWGnbqtcqWGnbqtaaGccqGHRaWkcqWGobGtdaqhaaWcbaGaem4zaCMaemyAaKMaemOAaOgabaGaemyta0Kaemyta0eaaOGaey4kaSIaeuOPdyKaem4uam1aa0baaSqaaiabdEgaNjabdMgaPjabdQgaQbqaaiabd2eanjabd2eanbaakiabcYcaSiabikdaYmaaCaaaleqabaGaeGymaeJaeGOnaydaaOGaeiykaKcaaa@5752@. The RMA algorithm [12] was used to summarize simulated probe-level data into a signal value, and the MAS5 algorithm [5] was used to obtain the detection p-value.

### Design of simulation study

The simulation design is shown in Table 7. We assumed two groups, A and B, with *N*_*A *_and *N*_*B *_arrays, respectively. *G *genes were generated, of them the proportion of expressed genes is *k*, and the proportion of differentially expressed genes is *d *of the G*k expressed genes. We set the number of up-and -down regulated genes to be the same in this study. Therefore, the G genes are divided into four groups: a non-expressed gene group where genes are not expressed across all arrays in group A and group B; a non-differentially expressed gene group where genes are expressed but not differentially between groups A and B; an up-regulated gene group where the mean gene expression of gene *g *in group B is larger by *δ*_*g *_than that in group A; and a down-regulated gene group where the mean gene expression of gene *g *in group A is larger by *δ*_*g *_than that in group B.

We ran five simulation models following the above design. The specific parameters used in the five models are: number of genes: 1000; sample size: 25 arrays in groups A and B, respectively (for a total of 50 arrays); number of probes within each probeset: 16; proportion of expressed genes: 0.5; proportion of differentially expressed genes: 0.1; signal detection variance: 0.25; multiplicative error variance: 0.05 and sensitivity parameter *ν*: 0.05.

### Duchenne muscular dystrophy data

Haslett et al. [16] hybridized the total RNA to HG-U95Av2 GeneChips. They used MAS5 to obtain signal intensities and normalized with a linear regression. Tests of differential expression were based on geometric fold change (GFC) [16]. The differential expression of 12 genes (13 probesets) was confirmed by quantitative RT-PCR analysis of seven DMD biopsies and four unaffected biopsies. We used only 23 arrays (only 11 DMD arrays) for our re-analysis since one file was truncated. Raw data were converted to signal estimates using both MAS5 [5] and RMA [12], both were implemented using the affy package in Bioconductor [34].

### Spiked-in data of Choe et al. [17]

The 'spiked-in' experiment (Choe et al. [17]) for Affymetrix Genechips provides a controlled dataset of 3,860 RNA species with known sequence and known concentration. Two different samples were prepared and hybridized in triplicate to Affymetric GeneChips; these are called the 'constant' (C) and 'spike' (S) samples. Out of 3,860 RNA species, 2,551 of them were created to have the same concentrations in both samples while the rest (1,309) were spiked in with different concentrations between the S and C samples. Ten fold-change levels, ranging from 1.2 to 4-fold, were assigned to the spiked-in RNAs. Basically, all the RNAs with positive log fold changes can be thought of as differentially expressed genes. In this study, we considered the top 1000 probesets as differentially expressed genes (as did Choe et al. [17]).

## Authors' contributions

CG initiated, designed and managed the study. CG also proposed the methods in this manuscript. JB participated in designing and managing the study. PH conducted data analysis and drafted the manuscript. CG and JB revised the manuscript. All authors read and approved the final manuscript.

## Figures and Tables

**Table 1:** Test statistics and quality measures. For the quality-based tests, the summarized quality measure, across treatment groups, is defined by *Q*_*g *_= max_*w*∈{1...*W*} _Rgw
 MathType@MTEF@5@5@+=feaafiart1ev1aaatCvAUfKttLearuWrP9MDH5MBPbIqV92AaeXatLxBI9gBaebbnrfifHhDYfgasaacH8akY=wiFfYdH8Gipec8Eeeu0xXdbba9frFj0=OqFfea0dXdd9vqai=hGuQ8kuc9pgc9s8qqaq=dirpe0xb9q8qiLsFr0=vr0=vr0dc8meaabaqaciaacaGaaeqabaqabeGadaaakeaacqWGsbGudaqhaaWcbaGaem4zaCgabaGaem4DaChaaaaa@30D4@.

**Quality Measures**	**Array specific measure**	**Group-specific measure **Rgw MathType@MTEF@5@5@+=feaafiart1ev1aaatCvAUfKttLearuWrP9MDH5MBPbIqV92AaeXatLxBI9gBaebbnrfifHhDYfgasaacH8akY=wiFfYdH8Gipec8Eeeu0xXdbba9frFj0=OqFfea0dXdd9vqai=hGuQ8kuc9pgc9s8qqaq=dirpe0xb9q8qiLsFr0=vr0=vr0dc8meaabaqaciaacaGaaeqabaqabeGadaaakeaacqWGsbGudaqhaaWcbaGaem4zaCgabaGaem4DaChaaaaa@30D4@	**Notes**
Qg0 MathType@MTEF@5@5@+=feaafiart1ev1aaatCvAUfKttLearuWrP9MDH5MBPbIqV92AaeXatLxBI9gBaebbnrfifHhDYfgasaacH8akY=wiFfYdH8Gipec8Eeeu0xXdbba9frFj0=OqFfea0dXdd9vqai=hGuQ8kuc9pgc9s8qqaq=dirpe0xb9q8qiLsFr0=vr0=vr0dc8meaabaqaciaacaGaaeqabaqabeGadaaakeaacqWGrbqudaqhaaWcbaGaem4zaCgabaGaeGimaadaaaaa@3049@	1.0	1.0	No filtering
Qgcall MathType@MTEF@5@5@+=feaafiart1ev1aaatCvAUfKttLearuWrP9MDH5MBPbIqV92AaeXatLxBI9gBaebbnrfifHhDYfgasaacH8akY=wiFfYdH8Gipec8Eeeu0xXdbba9frFj0=OqFfea0dXdd9vqai=hGuQ8kuc9pgc9s8qqaq=dirpe0xb9q8qiLsFr0=vr0=vr0dc8meaabaqaciaacaGaaeqabaqabeGadaaakeaacqWGrbqudaqhaaWcbaGaem4zaCgabaGaem4yamMaemyyaeMaemiBaWMaemiBaWgaaaaa@34B7@	qgiw*={1if call is P0otherwise MathType@MTEF@5@5@+=feaafiart1ev1aaatCvAUfKttLearuWrP9MDH5MBPbIqV92AaeXatLxBI9gBaebbnrfifHhDYfgasaacH8akY=wiFfYdH8Gipec8Eeeu0xXdbba9frFj0=OqFfea0dXdd9vqai=hGuQ8kuc9pgc9s8qqaq=dirpe0xb9q8qiLsFr0=vr0=vr0dc8meaabaqaciaacaGaaeqabaqabeGadaaakeaacqWGXbqCdaqhaaWcbaGaem4zaCMaemyAaKMaem4DaChabaGaeiOkaOcaaOGaeyypa0ZaaiqaaeaafaqabeGacaaabaGaeGymaedabaGaemyAaKMaemOzayMaaGPaVlabdogaJjabdggaHjabdYgaSjabdYgaSjaaykW7cqWGPbqAcqWGZbWCcaaMc8UaemiuaafabaGaeGimaadabaGaem4Ba8MaemiDaqNaemiAaGMaemyzauMaemOCaiNaem4DaCNaemyAaKMaem4CamNaemyzaugaaaGaay5Eaaaaaa@5486@	min⁡i∈{1…I}qgiw* MathType@MTEF@5@5@+=feaafiart1ev1aaatCvAUfKttLearuWrP9MDH5MBPbIqV92AaeXatLxBI9gBaebbnrfifHhDYfgasaacH8akY=wiFfYdH8Gipec8Eeeu0xXdbba9frFj0=OqFfea0dXdd9vqai=hGuQ8kuc9pgc9s8qqaq=dirpe0xb9q8qiLsFr0=vr0=vr0dc8meaabaqaciaacaGaaeqabaqabeGadaaakeaacyGGTbqBcqGGPbqAcqGGUbGBdaWgaaWcbaGaemyAaKMaeyicI4Saei4EaSNaeGymaeJaeSOjGSKaemysaKKaeiyFa0habeaakiabdghaXnaaDaaaleaacqWGNbWzcqWGPbqAcqWG3bWDaeaacqGGQaGkaaaaaa@40AD@	All arrays must have present call to be included
Qgmeanp MathType@MTEF@5@5@+=feaafiart1ev1aaatCvAUfKttLearuWrP9MDH5MBPbIqV92AaeXatLxBI9gBaebbnrfifHhDYfgasaacH8akY=wiFfYdH8Gipec8Eeeu0xXdbba9frFj0=OqFfea0dXdd9vqai=hGuQ8kuc9pgc9s8qqaq=dirpe0xb9q8qiLsFr0=vr0=vr0dc8meaabaqaciaacaGaaeqabaqabeGadaaakeaacqWGrbqudaqhaaWcbaGaem4zaCgabaGaemyBa0MaemyzauMaemyyaeMaemOBa4MaemiCaahaaaaa@362A@	*q*_*giw *_= detection p-value	1−q¯gw=1−∑i∈w(qgiw)/nw MathType@MTEF@5@5@+=feaafiart1ev1aaatCvAUfKttLearuWrP9MDH5MBPbIqV92AaeXatLxBI9gBaebbnrfifHhDYfgasaacH8akY=wiFfYdH8Gipec8Eeeu0xXdbba9frFj0=OqFfea0dXdd9vqai=hGuQ8kuc9pgc9s8qqaq=dirpe0xb9q8qiLsFr0=vr0=vr0dc8meaabaqaciaacaGaaeqabaqabeGadaaakeaacqaIXaqmcqGHsislcuWGXbqCgaqeamaaBaaaleaacqWGNbWzcqWG3bWDaeqaaOGaeyypa0JaeGymaeJaeyOeI0YaaabuaeaacqGGOaakcqWGXbqCdaWgaaWcbaGaem4zaCMaemyAaKMaem4DaChabeaakiabcMcaPiabc+caViabd6gaUnaaBaaaleaacqWG3bWDaeqaaaqaaiabdMgaPjabgIGiolabdEha3bqab0GaeyyeIuoaaaa@47CB@	*n*_*w *_is the number of arrays in group *w*
Qgexp⁡ MathType@MTEF@5@5@+=feaafiart1ev1aaatCvAUfKttLearuWrP9MDH5MBPbIqV92AaeXatLxBI9gBaebbnrfifHhDYfgasaacH8akY=wiFfYdH8Gipec8Eeeu0xXdbba9frFj0=OqFfea0dXdd9vqai=hGuQ8kuc9pgc9s8qqaq=dirpe0xb9q8qiLsFr0=vr0=vr0dc8meaabaqaciaacaGaaeqabaqabeGadaaakeaacqWGrbqudaqhaaWcbaGaem4zaCgabaGagiyzauMaeiiEaGNaeiiCaahaaaaa@338F@	*q*_*giw *_= detection p-value	exp⁡⌊λ^gwlog⁡ν⌋ MathType@MTEF@5@5@+=feaafiart1ev1aaatCvAUfKttLearuWrP9MDH5MBPbIqV92AaeXatLxBI9gBaebbnrfifHhDYfgasaacH8akY=wiFfYdH8Gipec8Eeeu0xXdbba9frFj0=OqFfea0dXdd9vqai=hGuQ8kuc9pgc9s8qqaq=dirpe0xb9q8qiLsFr0=vr0=vr0dc8meaabaqaciaacaGaaeqabaqabeGadaaakeaacyGGLbqzcqGG4baEcqGGWbaCdaGbdaqaaGGaciqb=T7aSzaajaWaaSbaaSqaaiabdEgaNjabdEha3bqabaGccyGGSbaBcqGGVbWBcqGGNbWzcqWF9oGBaiaawcp+caGL7Jpaaaa@4098@ for sensitivity parameter *ν*	λ^gw=−nw/∑i∈wlog⁡(qgiw) MathType@MTEF@5@5@+=feaafiart1ev1aaatCvAUfKttLearuWrP9MDH5MBPbIqV92AaeXatLxBI9gBaebbnrfifHhDYfgasaacH8akY=wiFfYdH8Gipec8Eeeu0xXdbba9frFj0=OqFfea0dXdd9vqai=hGuQ8kuc9pgc9s8qqaq=dirpe0xb9q8qiLsFr0=vr0=vr0dc8meaabaqaciaacaGaaeqabaqabeGadaaakeaacuaH7oaBgaqcamaaBaaaleaacqWGNbWzcqWG3bWDaeqaaOGaeyypa0JaeyOeI0IaemOBa42aaSbaaSqaaiabdEha3bqabaGccqGGVaWldaaeqbqaaiGbcYgaSjabc+gaVjabcEgaNbWcbaGaemyAaKMaeyicI4Saem4DaChabeqdcqGHris5aOGaeiikaGIaemyCae3aaSbaaSqaaiabdEgaNjabdMgaPjabdEha3bqabaGccqGGPaqkaaa@497C@
Qgbeta MathType@MTEF@5@5@+=feaafiart1ev1aaatCvAUfKttLearuWrP9MDH5MBPbIqV92AaeXatLxBI9gBaebbnrfifHhDYfgasaacH8akY=wiFfYdH8Gipec8Eeeu0xXdbba9frFj0=OqFfea0dXdd9vqai=hGuQ8kuc9pgc9s8qqaq=dirpe0xb9q8qiLsFr0=vr0=vr0dc8meaabaqaciaacaGaaeqabaqabeGadaaakeaacqWGrbqudaqhaaWcbaGaem4zaCgabaGaemOyaiMaemyzauMaemiDaqNaemyyaegaaaaa@34B7@	*q*_*giw *_= detection p-value	νa^gw MathType@MTEF@5@5@+=feaafiart1ev1aaatCvAUfKttLearuWrP9MDH5MBPbIqV92AaeXatLxBI9gBaebbnrfifHhDYfgasaacH8akY=wiFfYdH8Gipec8Eeeu0xXdbba9frFj0=OqFfea0dXdd9vqai=hGuQ8kuc9pgc9s8qqaq=dirpe0xb9q8qiLsFr0=vr0=vr0dc8meaabaqaciaacaGaaeqabaqabeGadaaakeaaiiGacqWF9oGBdaahaaWcbeqaaiqbdggaHzaajaWaaSbaaWqaaiabdEgaNjabdEha3bqabaaaaaaa@32EE@	a^gw=q¯gw/(1−q¯gw) MathType@MTEF@5@5@+=feaafiart1ev1aaatCvAUfKttLearuWrP9MDH5MBPbIqV92AaeXatLxBI9gBaebbnrfifHhDYfgasaacH8akY=wiFfYdH8Gipec8Eeeu0xXdbba9frFj0=OqFfea0dXdd9vqai=hGuQ8kuc9pgc9s8qqaq=dirpe0xb9q8qiLsFr0=vr0=vr0dc8meaabaqaciaacaGaaeqabaqabeGadaaakeaacuWGHbqygaqcamaaBaaaleaacqWGNbWzcqWG3bWDaeqaaOGaeyypa0JafmyCaeNbaebadaWgaaWcbaGaem4zaCMaem4DaChabeaakiabc+caViabcIcaOiabigdaXiabgkHiTiqbdghaXzaaraWaaSbaaSqaaiabdEgaNjabdEha3bqabaGccqGGPaqkaaa@3F94@
Qgwe MathType@MTEF@5@5@+=feaafiart1ev1aaatCvAUfKttLearuWrP9MDH5MBPbIqV92AaeXatLxBI9gBaebbnrfifHhDYfgasaacH8akY=wiFfYdH8Gipec8Eeeu0xXdbba9frFj0=OqFfea0dXdd9vqai=hGuQ8kuc9pgc9s8qqaq=dirpe0xb9q8qiLsFr0=vr0=vr0dc8meaabaqaciaacaGaaeqabaqabeGadaaakeaacqWGrbqudaqhaaWcbaGaem4zaCgabaGaem4DaCNaemyzaugaaaaa@3225@	*q*_*giw *_= detection p-value	N/A	Weighted means x¯gw MathType@MTEF@5@5@+=feaafiart1ev1aaatCvAUfKttLearuWrP9MDH5MBPbIqV92AaeXatLxBI9gBaebbnrfifHhDYfgasaacH8akY=wiFfYdH8Gipec8Eeeu0xXdbba9frFj0=OqFfea0dXdd9vqai=hGuQ8kuc9pgc9s8qqaq=dirpe0xb9q8qiLsFr0=vr0=vr0dc8meaabaqaciaacaGaaeqabaqabeGadaaakeaacuWG4baEgaqeamaaBaaaleaacqWGNbWzcqWG3bWDaeqaaaaa@3137@ and weighted standard deviations *S*_*gw*_
QgLPE MathType@MTEF@5@5@+=feaafiart1ev1aaatCvAUfKttLearuWrP9MDH5MBPbIqV92AaeXatLxBI9gBaebbnrfifHhDYfgasaacH8akY=wiFfYdH8Gipec8Eeeu0xXdbba9frFj0=OqFfea0dXdd9vqai=hGuQ8kuc9pgc9s8qqaq=dirpe0xb9q8qiLsFr0=vr0=vr0dc8meaabaqaciaacaGaaeqabaqabeGadaaakeaacqWGrbqudaqhaaWcbaGaem4zaCgabaGaemitaWKaemiuaaLaemyraueaaaaa@32B8@	N/A	N/A	Local pooled error test [18]

**Table 2:** Area under the curves (AUCs) for five simulation models *

Model	Multiple Testing	Quality Measures						
		
		Qg0 MathType@MTEF@5@5@+=feaafiart1ev1aaatCvAUfKttLearuWrP9MDH5MBPbIqV92AaeXatLxBI9gBaebbnrfifHhDYfgasaacH8akY=wiFfYdH8Gipec8Eeeu0xXdbba9frFj0=OqFfea0dXdd9vqai=hGuQ8kuc9pgc9s8qqaq=dirpe0xb9q8qiLsFr0=vr0=vr0dc8meaabaqaciaacaGaaeqabaqabeGadaaakeaacqWGrbqudaqhaaWcbaGaem4zaCgabaGaeGimaadaaaaa@3049@	Qgcall MathType@MTEF@5@5@+=feaafiart1ev1aaatCvAUfKttLearuWrP9MDH5MBPbIqV92AaeXatLxBI9gBaebbnrfifHhDYfgasaacH8akY=wiFfYdH8Gipec8Eeeu0xXdbba9frFj0=OqFfea0dXdd9vqai=hGuQ8kuc9pgc9s8qqaq=dirpe0xb9q8qiLsFr0=vr0=vr0dc8meaabaqaciaacaGaaeqabaqabeGadaaakeaacqWGrbqudaqhaaWcbaGaem4zaCgabaGaem4yamMaemyyaeMaemiBaWMaemiBaWgaaaaa@34B7@	Qgexp⁡ MathType@MTEF@5@5@+=feaafiart1ev1aaatCvAUfKttLearuWrP9MDH5MBPbIqV92AaeXatLxBI9gBaebbnrfifHhDYfgasaacH8akY=wiFfYdH8Gipec8Eeeu0xXdbba9frFj0=OqFfea0dXdd9vqai=hGuQ8kuc9pgc9s8qqaq=dirpe0xb9q8qiLsFr0=vr0=vr0dc8meaabaqaciaacaGaaeqabaqabeGadaaakeaacqWGrbqudaqhaaWcbaGaem4zaCgabaGagiyzauMaeiiEaGNaeiiCaahaaaaa@338F@	Qgbeta MathType@MTEF@5@5@+=feaafiart1ev1aaatCvAUfKttLearuWrP9MDH5MBPbIqV92AaeXatLxBI9gBaebbnrfifHhDYfgasaacH8akY=wiFfYdH8Gipec8Eeeu0xXdbba9frFj0=OqFfea0dXdd9vqai=hGuQ8kuc9pgc9s8qqaq=dirpe0xb9q8qiLsFr0=vr0=vr0dc8meaabaqaciaacaGaaeqabaqabeGadaaakeaacqWGrbqudaqhaaWcbaGaem4zaCgabaGaemOyaiMaemyzauMaemiDaqNaemyyaegaaaaa@34B7@	Qgmeanp MathType@MTEF@5@5@+=feaafiart1ev1aaatCvAUfKttLearuWrP9MDH5MBPbIqV92AaeXatLxBI9gBaebbnrfifHhDYfgasaacH8akY=wiFfYdH8Gipec8Eeeu0xXdbba9frFj0=OqFfea0dXdd9vqai=hGuQ8kuc9pgc9s8qqaq=dirpe0xb9q8qiLsFr0=vr0=vr0dc8meaabaqaciaacaGaaeqabaqabeGadaaakeaacqWGrbqudaqhaaWcbaGaem4zaCgabaGaemyBa0MaemyzauMaemyyaeMaemOBa4MaemiCaahaaaaa@362A@	Qgwe MathType@MTEF@5@5@+=feaafiart1ev1aaatCvAUfKttLearuWrP9MDH5MBPbIqV92AaeXatLxBI9gBaebbnrfifHhDYfgasaacH8akY=wiFfYdH8Gipec8Eeeu0xXdbba9frFj0=OqFfea0dXdd9vqai=hGuQ8kuc9pgc9s8qqaq=dirpe0xb9q8qiLsFr0=vr0=vr0dc8meaabaqaciaacaGaaeqabaqabeGadaaakeaacqWGrbqudaqhaaWcbaGaem4zaCgabaGaem4DaCNaemyzaugaaaaa@3225@	QgLPE MathType@MTEF@5@5@+=feaafiart1ev1aaatCvAUfKttLearuWrP9MDH5MBPbIqV92AaeXatLxBI9gBaebbnrfifHhDYfgasaacH8akY=wiFfYdH8Gipec8Eeeu0xXdbba9frFj0=OqFfea0dXdd9vqai=hGuQ8kuc9pgc9s8qqaq=dirpe0xb9q8qiLsFr0=vr0=vr0dc8meaabaqaciaacaGaaeqabaqabeGadaaakeaacqWGrbqudaqhaaWcbaGaem4zaCgabaGaemitaWKaemiuaaLaemyraueaaaaa@32B8@
Model 1 *δ*_*g *_= 2.5	Unadjusted	0.777	0.380	0.925	0.892	0.871	0.743	0.924
	WBH	0.812	0.382	0.929	0.895	0.885	0.772	0.932
Model 2 *δ*_*g *_= 2.0	Unadjusted	0.840	0.387	0.934	0.907	0.898	0.809	0.932
	WBH	0.878	0.387	0.937	0.912	0.911	0.846	0.937
Model 3 *δ*_*g *_= 1.5	Unadjusted	0.895	0.394	0.933	0.918	0.922	0.868	0.932
	WBH	0.925	0.395	0.937	0.923	0.932	0.902	0.936
Model 4 *δ*_*g *_= 1.0	Unadjusted	0.918	0.399	0.921	0.913	0.926	0.889	0.915
	WBH	0.935	0.401	0.917	0.918	0.934	0.912	0.891
Model 5 *δ*_*g *_= 0.5	Unadjusted	0.876	0.408	0.875	0.876	0.884	0.850	0.850
	WBH	0.882	0.410	0.858	0.861	0.882	0.860	0.770

For Qgexp⁡
 MathType@MTEF@5@5@+=feaafiart1ev1aaatCvAUfKttLearuWrP9MDH5MBPbIqV92AaeXatLxBI9gBaebbnrfifHhDYfgasaacH8akY=wiFfYdH8Gipec8Eeeu0xXdbba9frFj0=OqFfea0dXdd9vqai=hGuQ8kuc9pgc9s8qqaq=dirpe0xb9q8qiLsFr0=vr0=vr0dc8meaabaqaciaacaGaaeqabaqabeGadaaakeaacqWGrbqudaqhaaWcbaGaem4zaCgabaGagiyzauMaeiiEaGNaeiiCaahaaaaa@338F@ and Qgbeta
 MathType@MTEF@5@5@+=feaafiart1ev1aaatCvAUfKttLearuWrP9MDH5MBPbIqV92AaeXatLxBI9gBaebbnrfifHhDYfgasaacH8akY=wiFfYdH8Gipec8Eeeu0xXdbba9frFj0=OqFfea0dXdd9vqai=hGuQ8kuc9pgc9s8qqaq=dirpe0xb9q8qiLsFr0=vr0=vr0dc8meaabaqaciaacaGaaeqabaqabeGadaaakeaacqWGrbqudaqhaaWcbaGaem4zaCgabaGaemOyaiMaemyzauMaemiDaqNaemyyaegaaaaa@34B7@, the sensitivity parameter *ν *was set to 0.05.

* Simulated probe level data was summarized using RMA

**Table 3:** Agreement in probeset selections between our methods and Haslett et al. [16]: Given a chosen number of selected probesets, how many of the probesets selected by GFC were also selected by the methods in this paper (corresponding false discovery rate WBH-FDR)

Summarization Method	Number of probesets selected	Qg0 MathType@MTEF@5@5@+=feaafiart1ev1aaatCvAUfKttLearuWrP9MDH5MBPbIqV92AaeXatLxBI9gBaebbnrfifHhDYfgasaacH8akY=wiFfYdH8Gipec8Eeeu0xXdbba9frFj0=OqFfea0dXdd9vqai=hGuQ8kuc9pgc9s8qqaq=dirpe0xb9q8qiLsFr0=vr0=vr0dc8meaabaqaciaacaGaaeqabaqabeGadaaakeaacqWGrbqudaqhaaWcbaGaem4zaCgabaGaeGimaadaaaaa@3049@	Qgcall MathType@MTEF@5@5@+=feaafiart1ev1aaatCvAUfKttLearuWrP9MDH5MBPbIqV92AaeXatLxBI9gBaebbnrfifHhDYfgasaacH8akY=wiFfYdH8Gipec8Eeeu0xXdbba9frFj0=OqFfea0dXdd9vqai=hGuQ8kuc9pgc9s8qqaq=dirpe0xb9q8qiLsFr0=vr0=vr0dc8meaabaqaciaacaGaaeqabaqabeGadaaakeaacqWGrbqudaqhaaWcbaGaem4zaCgabaGaem4yamMaemyyaeMaemiBaWMaemiBaWgaaaaa@34B7@	Qgexp⁡e MathType@MTEF@5@5@+=feaafiart1ev1aaatCvAUfKttLearuWrP9MDH5MBPbIqV92AaeXatLxBI9gBaebbnrfifHhDYfgasaacH8akY=wiFfYdH8Gipec8Eeeu0xXdbba9frFj0=OqFfea0dXdd9vqai=hGuQ8kuc9pgc9s8qqaq=dirpe0xb9q8qiLsFr0=vr0=vr0dc8meaabaqaciaacaGaaeqabaqabeGadaaakeaacqWGrbqudaqhaaWcbaGaem4zaCgabaGagiyzauMaeiiEaGNaeiiCaa3aaSbaaWqaaiabbwgaLbqabaaaaaaa@350D@	Qgbetae MathType@MTEF@5@5@+=feaafiart1ev1aaatCvAUfKttLearuWrP9MDH5MBPbIqV92AaeXatLxBI9gBaebbnrfifHhDYfgasaacH8akY=wiFfYdH8Gipec8Eeeu0xXdbba9frFj0=OqFfea0dXdd9vqai=hGuQ8kuc9pgc9s8qqaq=dirpe0xb9q8qiLsFr0=vr0=vr0dc8meaabaqaciaacaGaaeqabaqabeGadaaakeaacqWGrbqudaqhaaWcbaGaem4zaCgabaGaemOyaiMaemyzauMaemiDaqNaemyyae2aaSbaaWqaaiabbwgaLbqabaaaaaaa@3635@	Qgmeanp MathType@MTEF@5@5@+=feaafiart1ev1aaatCvAUfKttLearuWrP9MDH5MBPbIqV92AaeXatLxBI9gBaebbnrfifHhDYfgasaacH8akY=wiFfYdH8Gipec8Eeeu0xXdbba9frFj0=OqFfea0dXdd9vqai=hGuQ8kuc9pgc9s8qqaq=dirpe0xb9q8qiLsFr0=vr0=vr0dc8meaabaqaciaacaGaaeqabaqabeGadaaakeaacqWGrbqudaqhaaWcbaGaem4zaCgabaGaemyBa0MaemyzauMaemyyaeMaemOBa4MaemiCaahaaaaa@362A@	Qgwe MathType@MTEF@5@5@+=feaafiart1ev1aaatCvAUfKttLearuWrP9MDH5MBPbIqV92AaeXatLxBI9gBaebbnrfifHhDYfgasaacH8akY=wiFfYdH8Gipec8Eeeu0xXdbba9frFj0=OqFfea0dXdd9vqai=hGuQ8kuc9pgc9s8qqaq=dirpe0xb9q8qiLsFr0=vr0=vr0dc8meaabaqaciaacaGaaeqabaqabeGadaaakeaacqWGrbqudaqhaaWcbaGaem4zaCgabaGaem4DaCNaemyzaugaaaaa@3225@	QgLPE MathType@MTEF@5@5@+=feaafiart1ev1aaatCvAUfKttLearuWrP9MDH5MBPbIqV92AaeXatLxBI9gBaebbnrfifHhDYfgasaacH8akY=wiFfYdH8Gipec8Eeeu0xXdbba9frFj0=OqFfea0dXdd9vqai=hGuQ8kuc9pgc9s8qqaq=dirpe0xb9q8qiLsFr0=vr0=vr0dc8meaabaqaciaacaGaaeqabaqabeGadaaakeaacqWGrbqudaqhaaWcbaGaem4zaCgabaGaemitaWKaemiuaaLaemyraueaaaaa@32B8@
MAS5	30^a^	10^b ^(3.6e-06^c^)	6 (7.6e-06)	10 (6.2e-04)	10 (4.9e-06)	10 (3.6e-06)	11 (3.6e-06)	17 (2.3e-21)
	50	21 (1.4e-05)	13 (1.6e-05)	25 (2.3e-03)	22 (1.0e-05)	21 (1.2e-05)	22 (7.8e-06)	32 (6.0e-13)
	100	56 (3.5e-05)	40 (1.5e-04)	59 (4.1e-03)	57 (2.2e-05)	56 (2.6e-05)	56 (1.9e-05)	61 (3.3e-09)
	139 (2.3e-03)	94 (1.1e-04)	65 (3.3e-04)	96 (9.2e-03)	95 (8.4e-05)	94 (9.3e-05)	94 (6.1e-05)	76 (4.1e-07)
RMA	30	8^d ^(1.3e-05)	7 (2.5e-05)	9 (1.9e-03)	8 (7.5e-06)	8 (9.7e-06)	8 (5.6e-06)	20 (1.8e-18)
	50	24 (4.3e-05)	13 (5.0e-05)	26 (3.4e-03)	24 (2.8e-05)	24 (3.2e-05)	24 (2.0e-05)	30 (1.8e-11)
	100	60 (1.4e-04)	42 (3.3e-04)	63 (1.1e-02)	60 (9.8e-05)	60 (1.3e-04)	59 (8.0e-05)	62 (3.5e-07)
	139	85 (2.5e-04)	69 (7.4e-04)	92 (1.9e-02)	92 (2.0e-04)	89 (2.2e-04)	85 (1.5e-04)	77 (2.2e-05)

^a ^The top 30 probesets were selected by each method.

^b ^10 probesets overlapped between the top 30 probesets selected by GFC and the top 30 probesets selected by Qg0
 MathType@MTEF@5@5@+=feaafiart1ev1aaatCvAUfKttLearuWrP9MDH5MBPbIqV92AaeXatLxBI9gBaebbnrfifHhDYfgasaacH8akY=wiFfYdH8Gipec8Eeeu0xXdbba9frFj0=OqFfea0dXdd9vqai=hGuQ8kuc9pgc9s8qqaq=dirpe0xb9q8qiLsFr0=vr0=vr0dc8meaabaqaciaacaGaaeqabaqabeGadaaakeaacqWGrbqudaqhaaWcbaGaem4zaCgabaGaeGimaadaaaaa@3049@, when data were summarized using MAS5.

^c ^The WBH-FDR for the top 30 probesets selected by Qg0
 MathType@MTEF@5@5@+=feaafiart1ev1aaatCvAUfKttLearuWrP9MDH5MBPbIqV92AaeXatLxBI9gBaebbnrfifHhDYfgasaacH8akY=wiFfYdH8Gipec8Eeeu0xXdbba9frFj0=OqFfea0dXdd9vqai=hGuQ8kuc9pgc9s8qqaq=dirpe0xb9q8qiLsFr0=vr0=vr0dc8meaabaqaciaacaGaaeqabaqabeGadaaakeaacqWGrbqudaqhaaWcbaGaem4zaCgabaGaeGimaadaaaaa@3049@ was 3.6e-06.

^d ^8 probesets overlapped between the top 30 probesets selected by GFC (data normalized by MAS5) and the top 30 probesets selected by Qg0
 MathType@MTEF@5@5@+=feaafiart1ev1aaatCvAUfKttLearuWrP9MDH5MBPbIqV92AaeXatLxBI9gBaebbnrfifHhDYfgasaacH8akY=wiFfYdH8Gipec8Eeeu0xXdbba9frFj0=OqFfea0dXdd9vqai=hGuQ8kuc9pgc9s8qqaq=dirpe0xb9q8qiLsFr0=vr0=vr0dc8meaabaqaciaacaGaaeqabaqabeGadaaakeaacqWGrbqudaqhaaWcbaGaem4zaCgabaGaeGimaadaaaaa@3049@ (data normalized by RMA).

^e ^Sensitivity parameter *ν *= 0.05.

**Table 4:** Comparison of our methods and Haslett et al. [16]. Identification of differentially-expressed probesets validated by RT-PCR: given a chosen number of probesets selected by GFC, and the number of probesets validated by RT-PCR within this set, how many of the RT-PCR probesets were selected by the methods in this paper.

Method	# of probesets selected: # of GFC selections validated by RT-PCR	Qg0 MathType@MTEF@5@5@+=feaafiart1ev1aaatCvAUfKttLearuWrP9MDH5MBPbIqV92AaeXatLxBI9gBaebbnrfifHhDYfgasaacH8akY=wiFfYdH8Gipec8Eeeu0xXdbba9frFj0=OqFfea0dXdd9vqai=hGuQ8kuc9pgc9s8qqaq=dirpe0xb9q8qiLsFr0=vr0=vr0dc8meaabaqaciaacaGaaeqabaqabeGadaaakeaacqWGrbqudaqhaaWcbaGaem4zaCgabaGaeGimaadaaaaa@3049@	Qgcall MathType@MTEF@5@5@+=feaafiart1ev1aaatCvAUfKttLearuWrP9MDH5MBPbIqV92AaeXatLxBI9gBaebbnrfifHhDYfgasaacH8akY=wiFfYdH8Gipec8Eeeu0xXdbba9frFj0=OqFfea0dXdd9vqai=hGuQ8kuc9pgc9s8qqaq=dirpe0xb9q8qiLsFr0=vr0=vr0dc8meaabaqaciaacaGaaeqabaqabeGadaaakeaacqWGrbqudaqhaaWcbaGaem4zaCgabaGaem4yamMaemyyaeMaemiBaWMaemiBaWgaaaaa@34B7@	Qgexp⁡e MathType@MTEF@5@5@+=feaafiart1ev1aaatCvAUfKttLearuWrP9MDH5MBPbIqV92AaeXatLxBI9gBaebbnrfifHhDYfgasaacH8akY=wiFfYdH8Gipec8Eeeu0xXdbba9frFj0=OqFfea0dXdd9vqai=hGuQ8kuc9pgc9s8qqaq=dirpe0xb9q8qiLsFr0=vr0=vr0dc8meaabaqaciaacaGaaeqabaqabeGadaaakeaacqWGrbqudaqhaaWcbaGaem4zaCgabaGagiyzauMaeiiEaGNaeiiCaa3aaSbaaWqaaiabbwgaLbqabaaaaaaa@350D@	Qgbetae MathType@MTEF@5@5@+=feaafiart1ev1aaatCvAUfKttLearuWrP9MDH5MBPbIqV92AaeXatLxBI9gBaebbnrfifHhDYfgasaacH8akY=wiFfYdH8Gipec8Eeeu0xXdbba9frFj0=OqFfea0dXdd9vqai=hGuQ8kuc9pgc9s8qqaq=dirpe0xb9q8qiLsFr0=vr0=vr0dc8meaabaqaciaacaGaaeqabaqabeGadaaakeaacqWGrbqudaqhaaWcbaGaem4zaCgabaGaemOyaiMaemyzauMaemiDaqNaemyyae2aaSbaaWqaaiabbwgaLbqabaaaaaaa@3635@	Qgmeanp MathType@MTEF@5@5@+=feaafiart1ev1aaatCvAUfKttLearuWrP9MDH5MBPbIqV92AaeXatLxBI9gBaebbnrfifHhDYfgasaacH8akY=wiFfYdH8Gipec8Eeeu0xXdbba9frFj0=OqFfea0dXdd9vqai=hGuQ8kuc9pgc9s8qqaq=dirpe0xb9q8qiLsFr0=vr0=vr0dc8meaabaqaciaacaGaaeqabaqabeGadaaakeaacqWGrbqudaqhaaWcbaGaem4zaCgabaGaemyBa0MaemyzauMaemyyaeMaemOBa4MaemiCaahaaaaa@362A@	Qgwe MathType@MTEF@5@5@+=feaafiart1ev1aaatCvAUfKttLearuWrP9MDH5MBPbIqV92AaeXatLxBI9gBaebbnrfifHhDYfgasaacH8akY=wiFfYdH8Gipec8Eeeu0xXdbba9frFj0=OqFfea0dXdd9vqai=hGuQ8kuc9pgc9s8qqaq=dirpe0xb9q8qiLsFr0=vr0=vr0dc8meaabaqaciaacaGaaeqabaqabeGadaaakeaacqWGrbqudaqhaaWcbaGaem4zaCgabaGaem4DaCNaemyzaugaaaaa@3225@	QgLPE MathType@MTEF@5@5@+=feaafiart1ev1aaatCvAUfKttLearuWrP9MDH5MBPbIqV92AaeXatLxBI9gBaebbnrfifHhDYfgasaacH8akY=wiFfYdH8Gipec8Eeeu0xXdbba9frFj0=OqFfea0dXdd9vqai=hGuQ8kuc9pgc9s8qqaq=dirpe0xb9q8qiLsFr0=vr0=vr0dc8meaabaqaciaacaGaaeqabaqabeGadaaakeaacqWGrbqudaqhaaWcbaGaem4zaCgabaGaemitaWKaemiuaaLaemyraueaaaaa@32B8@
MAS5	30^a ^: 8^b^	5^c^	1	5	5	5	5	4
	50 : 11	8	2	7	7	8	7	6
	100 : 12	11	3	10	10	11	10	11
	139 : 13	12	4	11	11	12	12	12
RMA	30 : N/A	2^d^	1	4	2	2	2	6
	50 : N/A	6	2	7	6	6	6	7
	100 : N/A	9	4	9	9	9	8	8
	139 : N/A	10	4	11	10	10	10	11

^*a *^The top 30 probesets were selected by each method.

^b ^8 probesets in the top 30 were validated by RT-PCR in Haslett et al. [16].

^c ^5 RT-PCR probesets were among the top 30 probesets selected by Qg0
 MathType@MTEF@5@5@+=feaafiart1ev1aaatCvAUfKttLearuWrP9MDH5MBPbIqV92AaeXatLxBI9gBaebbnrfifHhDYfgasaacH8akY=wiFfYdH8Gipec8Eeeu0xXdbba9frFj0=OqFfea0dXdd9vqai=hGuQ8kuc9pgc9s8qqaq=dirpe0xb9q8qiLsFr0=vr0=vr0dc8meaabaqaciaacaGaaeqabaqabeGadaaakeaacqWGrbqudaqhaaWcbaGaem4zaCgabaGaeGimaadaaaaa@3049@. The 5 RT-PCR probesets are among the 8 RT-PCR probesets selected by GFC.

^d ^2 RT-PCR probesets were among the top 30 probesets selected by Qg0
 MathType@MTEF@5@5@+=feaafiart1ev1aaatCvAUfKttLearuWrP9MDH5MBPbIqV92AaeXatLxBI9gBaebbnrfifHhDYfgasaacH8akY=wiFfYdH8Gipec8Eeeu0xXdbba9frFj0=OqFfea0dXdd9vqai=hGuQ8kuc9pgc9s8qqaq=dirpe0xb9q8qiLsFr0=vr0=vr0dc8meaabaqaciaacaGaaeqabaqabeGadaaakeaacqWGrbqudaqhaaWcbaGaem4zaCgabaGaeGimaadaaaaa@3049@ (data normalized by RMA). The 2 RT-PCR probesets are among the 8 RT-PCR probesets selected by GFC, using the MAS5 normalization.

^e ^Sensitivity parameter *ν *= 0.05.

**Table 5:** Area under the curves (AUCs) of Choe's spiked-in data (*ν *= 0.05)

Method	Multiple Testing	Quality Measures						
		
		Qg0 MathType@MTEF@5@5@+=feaafiart1ev1aaatCvAUfKttLearuWrP9MDH5MBPbIqV92AaeXatLxBI9gBaebbnrfifHhDYfgasaacH8akY=wiFfYdH8Gipec8Eeeu0xXdbba9frFj0=OqFfea0dXdd9vqai=hGuQ8kuc9pgc9s8qqaq=dirpe0xb9q8qiLsFr0=vr0=vr0dc8meaabaqaciaacaGaaeqabaqabeGadaaakeaacqWGrbqudaqhaaWcbaGaem4zaCgabaGaeGimaadaaaaa@3049@	Qgcall MathType@MTEF@5@5@+=feaafiart1ev1aaatCvAUfKttLearuWrP9MDH5MBPbIqV92AaeXatLxBI9gBaebbnrfifHhDYfgasaacH8akY=wiFfYdH8Gipec8Eeeu0xXdbba9frFj0=OqFfea0dXdd9vqai=hGuQ8kuc9pgc9s8qqaq=dirpe0xb9q8qiLsFr0=vr0=vr0dc8meaabaqaciaacaGaaeqabaqabeGadaaakeaacqWGrbqudaqhaaWcbaGaem4zaCgabaGaem4yamMaemyyaeMaemiBaWMaemiBaWgaaaaa@34B7@	Qgexp⁡ MathType@MTEF@5@5@+=feaafiart1ev1aaatCvAUfKttLearuWrP9MDH5MBPbIqV92AaeXatLxBI9gBaebbnrfifHhDYfgasaacH8akY=wiFfYdH8Gipec8Eeeu0xXdbba9frFj0=OqFfea0dXdd9vqai=hGuQ8kuc9pgc9s8qqaq=dirpe0xb9q8qiLsFr0=vr0=vr0dc8meaabaqaciaacaGaaeqabaqabeGadaaakeaacqWGrbqudaqhaaWcbaGaem4zaCgabaGagiyzauMaeiiEaGNaeiiCaahaaaaa@338F@	Qgbeta MathType@MTEF@5@5@+=feaafiart1ev1aaatCvAUfKttLearuWrP9MDH5MBPbIqV92AaeXatLxBI9gBaebbnrfifHhDYfgasaacH8akY=wiFfYdH8Gipec8Eeeu0xXdbba9frFj0=OqFfea0dXdd9vqai=hGuQ8kuc9pgc9s8qqaq=dirpe0xb9q8qiLsFr0=vr0=vr0dc8meaabaqaciaacaGaaeqabaqabeGadaaakeaacqWGrbqudaqhaaWcbaGaem4zaCgabaGaemOyaiMaemyzauMaemiDaqNaemyyaegaaaaa@34B7@	Qgmeanp MathType@MTEF@5@5@+=feaafiart1ev1aaatCvAUfKttLearuWrP9MDH5MBPbIqV92AaeXatLxBI9gBaebbnrfifHhDYfgasaacH8akY=wiFfYdH8Gipec8Eeeu0xXdbba9frFj0=OqFfea0dXdd9vqai=hGuQ8kuc9pgc9s8qqaq=dirpe0xb9q8qiLsFr0=vr0=vr0dc8meaabaqaciaacaGaaeqabaqabeGadaaakeaacqWGrbqudaqhaaWcbaGaem4zaCgabaGaemyBa0MaemyzauMaemyyaeMaemOBa4MaemiCaahaaaaa@362A@	Qgwe MathType@MTEF@5@5@+=feaafiart1ev1aaatCvAUfKttLearuWrP9MDH5MBPbIqV92AaeXatLxBI9gBaebbnrfifHhDYfgasaacH8akY=wiFfYdH8Gipec8Eeeu0xXdbba9frFj0=OqFfea0dXdd9vqai=hGuQ8kuc9pgc9s8qqaq=dirpe0xb9q8qiLsFr0=vr0=vr0dc8meaabaqaciaacaGaaeqabaqabeGadaaakeaacqWGrbqudaqhaaWcbaGaem4zaCgabaGaem4DaCNaemyzaugaaaaa@3225@	QgLPE MathType@MTEF@5@5@+=feaafiart1ev1aaatCvAUfKttLearuWrP9MDH5MBPbIqV92AaeXatLxBI9gBaebbnrfifHhDYfgasaacH8akY=wiFfYdH8Gipec8Eeeu0xXdbba9frFj0=OqFfea0dXdd9vqai=hGuQ8kuc9pgc9s8qqaq=dirpe0xb9q8qiLsFr0=vr0=vr0dc8meaabaqaciaacaGaaeqabaqabeGadaaakeaacqWGrbqudaqhaaWcbaGaem4zaCgabaGaemitaWKaemiuaaLaemyraueaaaaa@32B8@
RMA	Unadjust	0.800	0.881	0.885	0.882	0.850	0804	0.854
	WBH	0.779	0.871	0.874	0.868	0.832	0.776	0.847
MAS5	Unadjust	0.815	0.889	0.901	0.896	0.869	0.814	0.922
	WBH	0.800	0.877	0.895	0.884	0.856	0.789	0.653

**Table 6:** Sensitivity and specificity for detecting differentially expressed genes, as a function of the sensitivity parameter *ν*

*ν*	Qgexp⁡ MathType@MTEF@5@5@+=feaafiart1ev1aaatCvAUfKttLearuWrP9MDH5MBPbIqV92AaeXatLxBI9gBaebbnrfifHhDYfgasaacH8akY=wiFfYdH8Gipec8Eeeu0xXdbba9frFj0=OqFfea0dXdd9vqai=hGuQ8kuc9pgc9s8qqaq=dirpe0xb9q8qiLsFr0=vr0=vr0dc8meaabaqaciaacaGaaeqabaqabeGadaaakeaacqWGrbqudaqhaaWcbaGaem4zaCgabaGagiyzauMaeiiEaGNaeiiCaahaaaaa@338F@		Qgbeta MathType@MTEF@5@5@+=feaafiart1ev1aaatCvAUfKttLearuWrP9MDH5MBPbIqV92AaeXatLxBI9gBaebbnrfifHhDYfgasaacH8akY=wiFfYdH8Gipec8Eeeu0xXdbba9frFj0=OqFfea0dXdd9vqai=hGuQ8kuc9pgc9s8qqaq=dirpe0xb9q8qiLsFr0=vr0=vr0dc8meaabaqaciaacaGaaeqabaqabeGadaaakeaacqWGrbqudaqhaaWcbaGaem4zaCgabaGaemOyaiMaemyzauMaemiDaqNaemyyaegaaaaa@34B7@	
	
	Sensitivity	Specificity	Sensitivity	Specificity
0.6	0.9996	0.7562	0.9998	0.7207
0.5	0.9995	0.7763	0.9997	0.7372
0.4	0.9981	0.7930	0.9994	0.7490
0.3	0.9967	0.8070	0.9990	0.7590
0.2	0.9922	0.8211	0.9984	0.7668
0.1	0.9831	0.8373	0.9956	0.7752
0.05	0.9660	0.8507	0.9936	0.7812
0.01	0.9167	0.8665	0.9413	0.7873

**Table 7:** Simulation structure of Affymetrix microarray data

		Group A			Group B		
		
		Array 1	......	Array *N*_*A*_	Array 1	......	Array *N*_*B*_
Up-regulated Gene Group	*g*_1_	*γ*_*g*_			*γ*_*g *_= *γ*_*g *_+ *δ*_*g*_		
	...						
	*g*_d*k*G/2_						
Down-regulated Gene Group	*g*_d*k*G/2+1_	*γ*_*g *_= *γ*_*g *_+ *δ*_*g*_			*γ*_*g*_		
	...						
	*g*_d*k*G_						
Non-differentially Expressed Gene Group	*g*_d*k*G+1_	*γ*_*g *_≠ 0 *δ*_*g *_= 0					
	...						
	*g*_k*G_						
Non- Expressed Gene Group	*g*_k*G+1_	*γ*_*g *_= 0 *δ*_*g *_= 0					
	...						
	*G*						
